# C3G/Rapgef1 Is Required in Multipolar Neurons for the Transition to a Bipolar Morphology during Cortical Development

**DOI:** 10.1371/journal.pone.0154174

**Published:** 2016-04-25

**Authors:** Bhavin Shah, Daniela Lutter, Magdalena L. Bochenek, Katsuhiro Kato, Yaroslav Tsytsyura, Natalia Glyvuk, Akira Sakakibara, Jürgen Klingauf, Ralf H. Adams, Andreas W. Püschel

**Affiliations:** 1 Institut für Molekulare Zellbiologie, Westfälische Wilhelms-Universität, Schloßplatz 5, D-48149 Münster, Germany; 2 Max-Planck-Institute for Molecular Biomedicine, Department of Tissue Morphogenesis, and University of Münster, Faculty of Medicine, D-48149 Münster, Germany; 3 Institute of Medical Physics and Biophysics, Robert-Koch Straße 31, D-48149 Münster, Germany; 4 Cells-in-Motion Cluster of Excellence, University of Münster, D-48149 Münster, Germany; 5 College of Life and Health Sciences, Chubu University, Kasugai 487–8501, Japan; Virginia Tech Carilion Research Institute, UNITED STATES

## Abstract

The establishment of a polarized morphology is essential for the development and function of neurons. During the development of the mammalian neocortex, neurons arise in the ventricular zone (VZ) from radial glia cells (RGCs) and leave the VZ to generate the cortical plate (CP). During their migration, newborn neurons first assume a multipolar morphology in the subventricular zone (SVZ) and lower intermediate zone (IZ). Subsequently, they undergo a multi-to-bipolar (MTB) transition to become bipolar in the upper IZ by developing a leading process and a trailing axon. The small GTPases Rap1A and Rap1B act as master regulators of neural cell polarity in the developing mouse neocortex. They are required for maintaining the polarity of RGCs and directing the MTB transition of multipolar neurons. Here we show that the Rap1 guanine nucleotide exchange factor (GEF) C3G (encoded by the *Rapgef1* gene) is a crucial regulator of the MTB transition *in vivo* by conditionally inactivating the *Rapgef1* gene in the developing mouse cortex at different time points during neuronal development. Inactivation of C3G results in defects in neuronal migration, axon formation and cortical lamination. Live cell imaging shows that C3G is required in cortical neurons for both the specification of an axon and the initiation of radial migration by forming a leading process.

## Introduction

The complex structure of the mammalian cortex depends on the precise control of the polarization and migration of neural cells. Cortical neurons arise in the VZ from radial glia cells (RGCs) that generate successive waves of neurons, which leave the VZ to generate the CP [1]. The first wave of postmitotic neurons forms the transient preplate (PP) that is split by the following wave of neurons into the marginal zone (MZ) and the subplate (SP) [2]. During their migration, newborn neurons first assume a multipolar morphology in the SVZ and lower IZ [3–6]. Subsequently, they undergo a multi-to-bipolar (MTB) transition to become bipolar in the upper IZ by developing a leading process that will become the apical dendrite and a trailing process that extends as the axon in the IZ [7–10]. Neurons migrate into the CP along the basal processes of the RGCs that span the cortex. When they approach the MZ, neurons attach the leading process to the MZ and translocate the soma to its final position by RGC-independent terminal translocation [4]. Successive divisions generate neurons that migrate past older ones to generate the six layers of the cortex in an inside-out pattern [11]. This process depends on the extracellular matrix protein reelin that is produced by the Cajal-Retzius (CR) cells located in the MZ [12, 13]. Downstream of the reelin receptors, Dab1 and Crk/CrkL activate the Rap1 GEF C3G followed by Rap1 GTPase activation [14–18]. Loss of reelin in reeler mice results in the failure to split the PP and an inverted organization of cortical layers due to the failure of later born neurons to migrate past older neurons while the formation of axons is not affected [19]. Reelin also regulates inside-out integrin-mediated adhesion during terminal translocation through C3G and Rap1 [20].

The MTB transition is a crucial step during neuronal differentiation and depends on both intrinsic and extrinsic factors [8, 9, 21]. One important factor that directs the MTB is N-cadherin, which in turn is regulated by Rap1 GTPases [22]. The Rap1 GTPases are essential for the establishment of neuronal polarity in cultured hippocampal neurons [23] and *in vivo* [24]. Our analysis of knockout mice shows that Rap1 GTPases are required for the MTB transition *in vivo* [24]. Their inactivation blocks the formation of an axon and a leading process. However, the factors that act upstream of Rap1 GTPases *in vivo* to initiate axon formation remain to be explored. One step towards the identification of these factors is to elucidate which Rap1 GEF is required *in vivo* for the MTB transition. Two different Rap1 GEFs have been implicated in this process. A hypomorphic *Rapgef1* allele that shows embryonic lethality starting at E14.5 [25] arrests neurons in the multipolar phase [26]. However, it was not investigated if C3G plays a role in axon formation and if it functions in RGCs or multipolar neurons. A more recent study showed that PDZ-GEF1 (Rapgef2) that shows a different domain organization than C3G is required for neuronal polarization during neocortical development [27]. A conditional knockout of *Rapgef2* in neuronal progenitors using *Emx1-Cre* results in the development of a disorganized, heterotopic cortex beneath a normally structured but thinner homotopic cortex while axon formation was not affected [28].

Here we investigate the function of the Rap1 GEF C3G in neuronal polarity *in vivo* using conditional knockouts. Our results show that C3G is required cell-autonomously in multipolar neurons for the MTB transition and the development of the cortical layers. The specification of axons and the initiation of radial migration by forming a leading process are blocked after deletion of *Rapgef1* in the developing embryonic cortex. Together, these defects result in the loss of axons and defects in cortical lamination.

## Results

### C3G is required for cortical development

**Fig 1:**
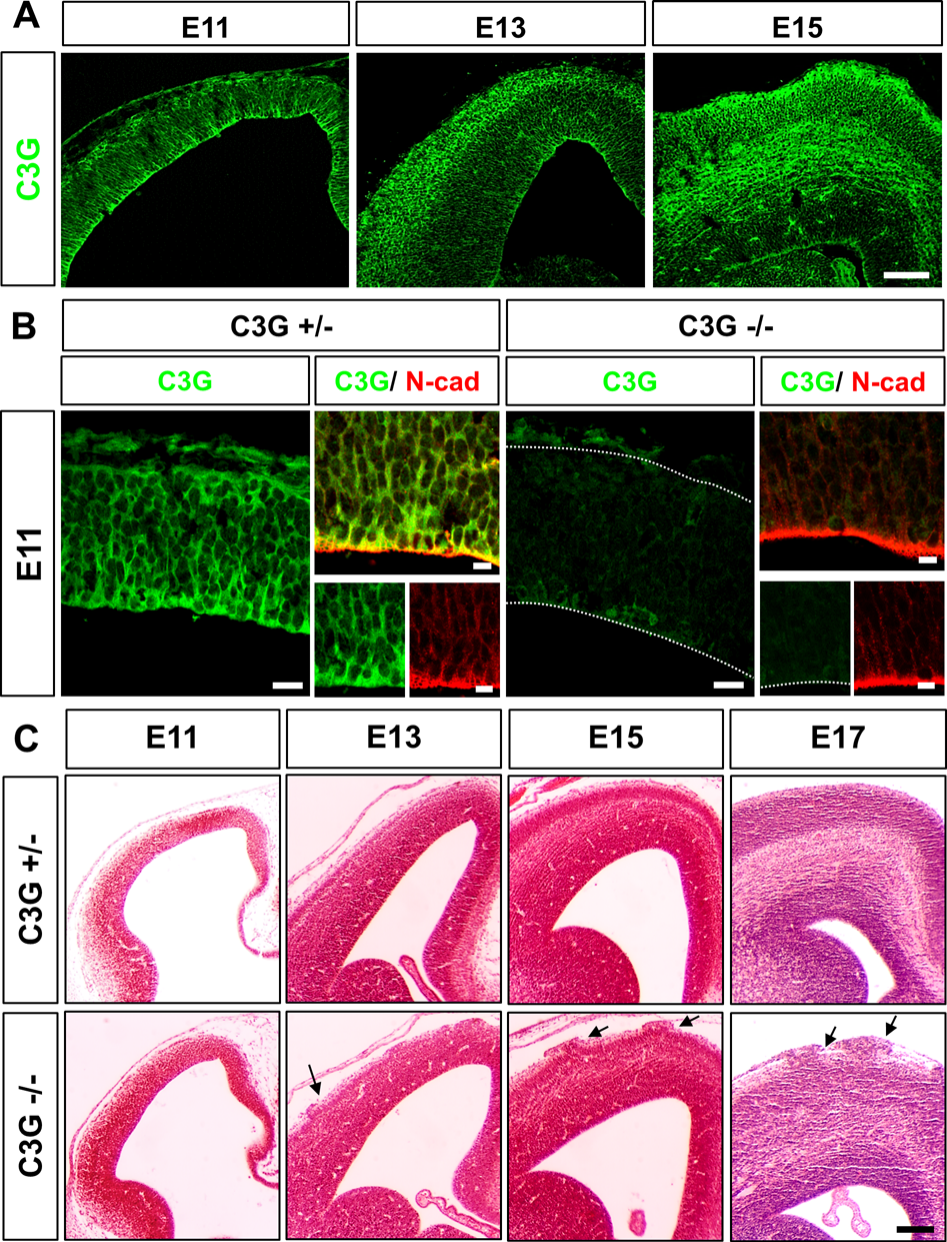
Cortical development is disrupted in the C3G^Emx1-KO^. (A) Coronal sections from the brain of wild type E11, E13 or E15 embryos were stained with an anti-C3G antibody. Note the apical enrichment of C3G immunoreactivity at E11 and an enhanced signal in the cortical plate at E13 and E15. (B) Coronal rostral sections from the brains of heterozygous (+/-) or homozygous (-/-) E11 C3G^Emx1-KO^ embryos were stained with anti-C3G (green) and anti-N-cadherin (red) antibodies. In the heterozygous controls, the C3G enrichment at the apical surface colocalizes with N-cadherin. Loss of C3G is evident from the loss of C3G immunoreactivity in the C3G^Emx1-KO^ at E11, however does not affect the levels of N-cadherin. Single confocal planes are shown. (C) Haematoxylin/eosin staining of coronal sections from the rostral brain shows defects in cortical lamination in the C3G^Emx1-KO^ cortices beginning at E13 compared to the heterozygous controls (+/-:*C3G*^flox/+^;*Emx1*^cre/+^). Arrows indicate neuronal ectopias. Dorsal is to the top and medial to the right. Scale bars are 100 μm (A, B) and 20 μm and 10 μm (C and inset). Images are representative for 3 independent experiments with 3 embryos per genotype from different litters.

To investigate if the Rap1 guanine nucleotide exchange factor (GEF) C3G (encoded by *Rapgef1*) is required for neuronal polarization we generated a conditional *Rapgef1* knockout (S1 Fig) to avoid the early embryonic lethality of the hypomorphic *Rapgef1* mutant [25]. An anti-C3G antibody against the N-terminal 300 amino acids detected C3G throughout the cortex and showed an enrichment at the apical surface at E11 (Fig 1A). C3G immunoreactivity increased when the neuronal layers were forming at E13 and E15 at the basal surface. By E15, C3G staining was also present in axonal fibers in the IZ. *Rapgef1* was specifically inactivated in neuronal progenitors of the cortex by crossing the conditional mutant with the *Emx1-Cr*e line that mediates a cortex-specific knockout in neural progenitors beginning at embryonic day E9.5 [29]. Staining of the embryonic cortex from homozygous E11 *Rapgef1*^flox/flox^;*Emx1*^Cre/+^ (called C3G^Emx1-KO^ hereafter) knockout embryos confirmed the loss of C3G as early as E11 (Fig 1B). To define the time point at which the defects start to appear we analyzed sections from C3G^Emx1-KO^ embryos by Hematoxylin-Eosin (HE) staining. Severe defects in cortical lamination that became obvious in homozygous C3G^Emx1-KO^ embryos at E13 (Fig 1C). The VZ was comparable to that from heterozygous or wild type brains but a separation into SVZ, IZ, and CP was no longer apparent. In addition, the cortex showed neuronal ectopias in the MZ from E13 onwards (Fig 1C). This phenotype is typical for a cobblestone lissencephaly where neurons migrate through ruptures of the basement membrane (BM) into the subarachnoid space [30]. The phenotype of the C3G^Emx1-KO^ is consistent with that described for the hypomorphic gene-trap allele that could be analyzed only until E14.5 [26]. Staining with an anti-Tbr2 antibody as a marker for intermediate progenitors (S2A and S2B Fig) did not reveal any difference between the heterozygous control and C3G^Emx1-KO^ cortex at the peak of intermediate progenitor numbers (E13) [24]. These results show that C3G plays an important role in establishing cortical architecture during development.

**Fig 2:**
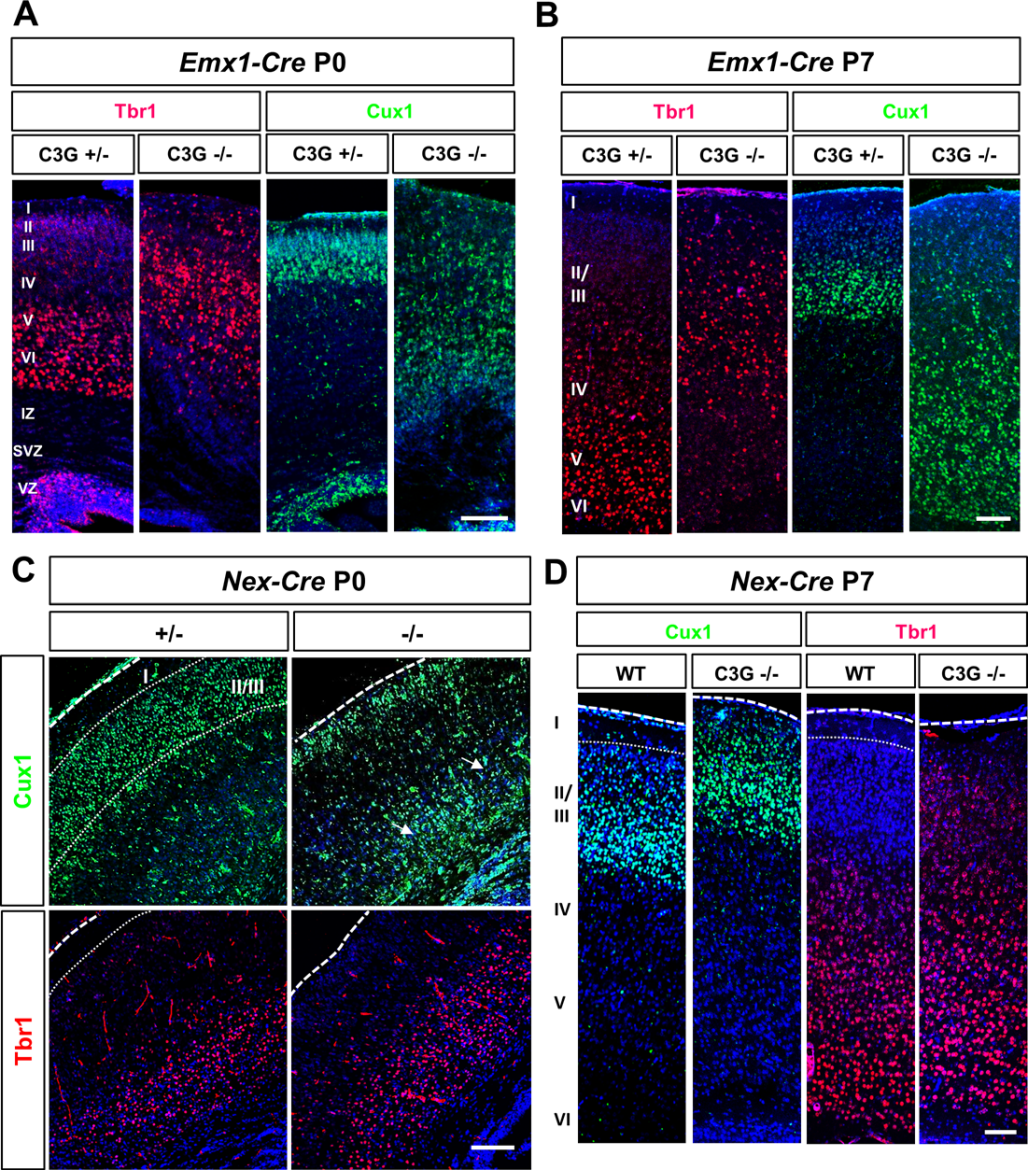
The loss of C3G leads to defects in cortical lamination. (A-D) Coronal sections from the brains of P0 (A, C) or P7 (B, D) C3G^Emx1-KO^ or C3G^Nex-KO^ embryos with the indicated genotypes were stained with antibodies for Tbr1 (layer V/VI and SP; red) and Cux1 (layers II/III, green). Both markers revealed a severe disruption of cortical organization in the C3G^Emx1-KO^. (B) Staining for Tbr1 and Cux1 at P7 demonstrates that the cortical plate is inverted in the C3G^Emx1-KO^. (C, D) No severe defects were observed in C3G^Nex-KO^, except for the presence of ectopic Cux1^+^ cells in deep layers at P0 (marked by arrows) and the invasion of Cux1^+^ cells into layer I. Dorsal is to the top. Single confocal planes are shown. Scale bars are 100 μm. Images are representative for 3 independent experiments with 3 embryos per genotype from different litters.

To analyze the defects in cortical lamination in more detail, we used the layer-specific markers Tbr1 (deeper layers V/VI and SP) and Cux1 (superficial layers II and III) at postnatal stages when neuronal migration is almost complete (P0) and the different layers are well established (P7). Tbr1^+^- and Cux1^+^ cells were not restricted to deep and superficial layers, respectively, but distributed throughout the P0 C3G^Emx1-KO^ cortex including neuronal ectopias at the pial surface (Fig 2A). These defects persisted at P7, when it also became obvious that the organization of neurons in the cortical plate was inverted in C3G^Emx1-KO^ brains (Fig 2B), a defect similar to the reeler phenotype [19]. Staining with antibodies for chondroitin sulfate proteoglycan (CSPG) and calretinin as markers for the MZ and SP confirmed the disorganization of cortical layers in the C3G^Emx1-KO^ cortex at E17 (S2C Fig). It also showed that the loss of C3G leads to defects in PP splitting as reported before [26]. However, some calretinin^+^ and CSPG^+^ cells were detectable in deeper layers in the C3G^Emx1-KO^, indicating an incomplete splitting of the PP.

The *Emx1-Cre* mediated knockout removes C3G in RGCs and the neurons generated by them. When we deleted *Rapgef1* in postmitotic neurons using the *Nex*-*Cre* line (called C3G^Nex-KO^ hereafter) to delete them specifically in postmitotic neurons [31] (S3A Fig). The formation of cortical layers was largely normal. The C3G^Nex-KO^ cortex showed only a defect in the migration of superficial layer neurons. The Cux1^+^ layers II and III were reduced at P0 and Cux1^+^ cells were scattered throughout the cortex and had invaded layer I (Fig 2C, 2D and S3B Fig). This defect was prominent in the C3G^Nex-KO^ at P7 where Cux1^+^ cells overmigrated into layer I (Fig 2D). These results show that C3G is essential for the formation of cortical layers and the migration of superficial layer neurons in the CP.

### C3G is required for integrin-mediated adhesions at the pial surface

**Fig 3:**
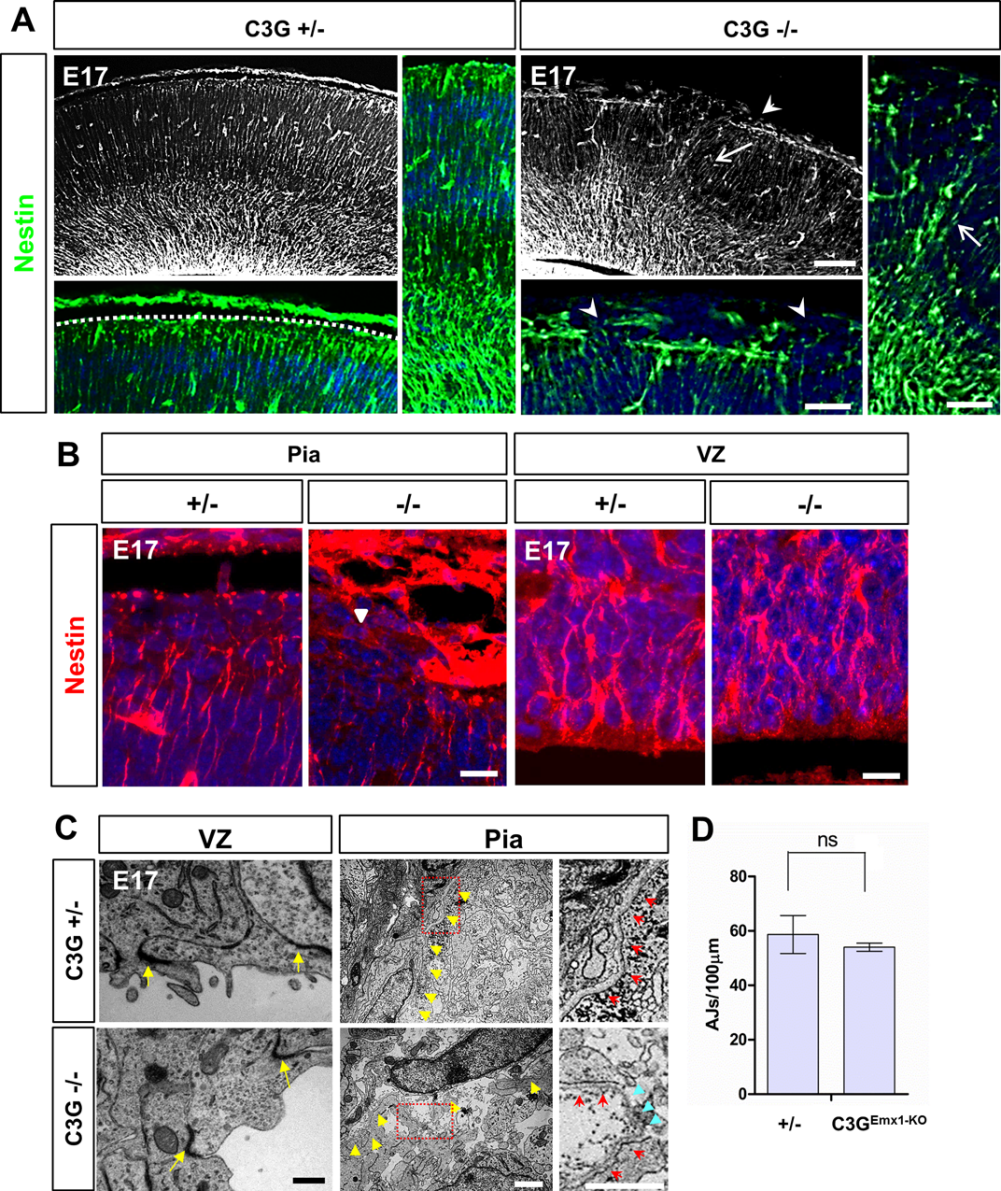
C3G^Emx1-KO^ shows defects in RGCs at the pial surface. (A, B) Coronal sections of C3G^Emx1-KO^ heterozygous or homozygous knockout embryos were stained with an anti-nestin antibody (green (A) or red (B)) and Hoechst 33342 (blue) at E17. (A) Higher magnification images from the pial surface show a continuous arrangement of glial fibers and endfeet in the heterozygous control compared to the disrupted organization of glial fibers and a rupture of basement membrane in the C3G^Emx1-KO^ cortex (arrowheads). Arrows indicate the disorganized glial fiber network. No defects at the VZ were seen in C3G^Emx1-KO^ (B). Single confocal planes are shown. (C, D) Coronal sections from the C3G^Emx1-KO^ cortex were analyzed by electron microscopy at E17. Images of the pial surface show a distinct BM in heterozygous embryos (yellow arrowheads) with a compact arrangement of cells below the BM (C). The BM appears to be ruptured in the C3G^Emx1-KO^ cortex and the cells protrude outside. The area marked by a box is shown at a higher magnification on the right depicting the BM. The red arrows (magnified panels) mark the continuous BM. The broken BM in the C3G^Emx1-KO^ pial surface is marked by blue arrowheads. No defects were seen at the VZ and the AJs formed normally (C). (D) The number of AJs per 100 μm of the VZ was quantified and no significant differences were found between the heterozygous and homozygous C3G^Emx1-KO^ embryos (n = 3 embryos per genotype, means ± s.e.m., ns, not significant, Student’s t-test). Scale bars are 100μm (A) 50μm (magnified panels in A), 10 μm (B), 500 nm (C, VZ) and 2 μm (C and magnified panels, Pia). Images are representative for 3 independent experiments with 3 embryos per genotype from different litters.

The phenotype of the C3G^Emx1-KO^ cortex (Fig 1B) resembles the defects in conditional mutants for *Itgb1* and *Ptk2* [32, 33]. Staining with an anti-nestin antibody as a marker for RGC revealed defects in the attachment of RGCs to the pial surface (Fig 3A and 3B). Nestin-positive RGC processes appear less organized and are not anchored to the BM in the C3G^Emx1-KO^ cortex in contrast to the regular organization of the RGC scaffold in heterozygous mutants at E17 (S4 Fig). However, no defects were observed in the VZ (Fig 3B) and staining with anti-N-cadherin antibodies did not reveal defects in adherens junctions (AJs) at the apical surface (Fig 1B). Thus, AJs are not affected in the C3G^Emx1-KO^ cortex unlike the severe defects seen in *Rap1a*^f/f^;*Rap1b*^f/f^;*Emx1*^Cre/+^ knockout mice [24]. Analysis of the VZ at the ultrastructural level by electron microscopy (EM) also did not detect defects in AJs in the C3G^Emx1-KO^ VZ, which displayed electron dense AJs throughout the apical surface (Fig 3C and 3D). By contrast, the EM analysis of the C3G^Emx1-KO^ cortex revealed severe defects in the cellular organization at the pial surface near the BM. The BM was interrupted and cells protruded through the BM, in C3G ^Emx1-KO^ brains (Fig 3C), consistent with the presence of ectopias.

**Fig 4:**
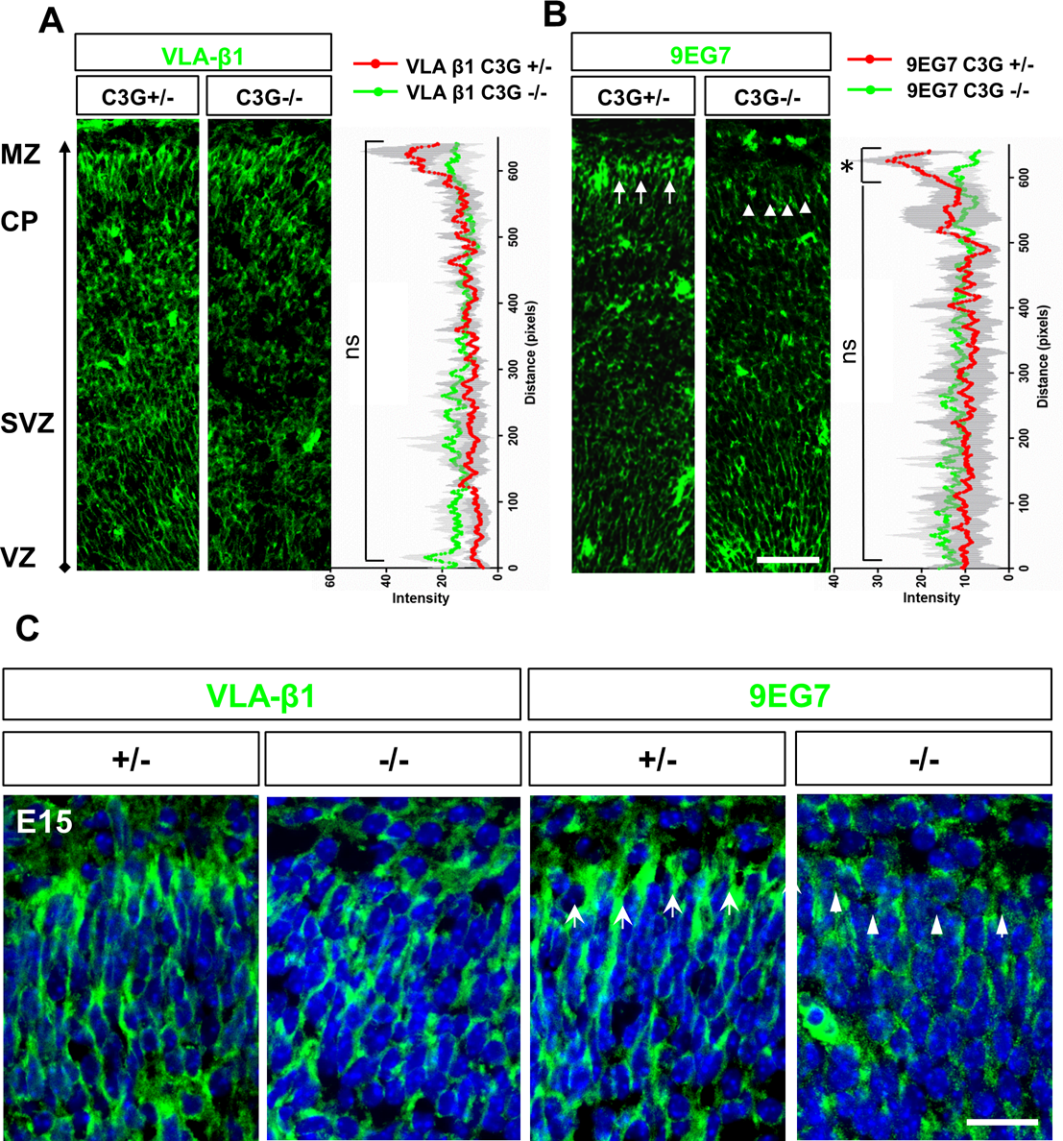
Loss of active β1 integrin in the C3G^Emx1-KO^ cortex. (A,-C) Coronal sections from heterozygous or homozygous E15 C3G^Emx1-KO^ cortex were stained with an anti- integrin β1 (VLA, green) and an antibody specific for active form of β1 integrin (9EG7, green) (A). No significant differences were found in the expression of β1 integrin when intensity values were plotted for the control and the C3G^Emx1-KO^. (B) The level of active β1 integrin was reduced at the pial surface in the C3G^Emx1-KO^ cortex (+/-: arrows, -/-: arrowheads). Intensity profiles of 9EG7 immunofluorescence signals (arbitrary units) measured from the VZ (bottom) to the pial surface (top) show a significant reduction in the intensity of 9EG7 signals at the pial surface in the C3G^Emx1-KO^ mutant cortex only in the MZ (in the last 40 pixel positions that include the glial endfeet) where active β1 integrins are enriched in control sections [20]. The fluorescence intensity values (arbitrary units) for staining with the VLA and 9EG7 antibodies were quantified at each pixel position along the ventricular to pial axis in a rectangular box comprising an area from the VZ to the MZ in sections from 3 different embryos per genotype. The significance of differences was calculated between means at each pixel position (means ± SEM, Student’s t-test to measure the difference in the means, *p ≤ 0.05; ns, not significant). (C) Higher magnification images of the pial surface stained with the above mentioned antibodies show defects in the 9EG7 staining in the C3G^Emx1-KO^. Dorsal is to the top. Single confocal planes are shown. Scale bar is 50 μm (A, B) and 20 μm (C).

C3G is known to regulate integrin function through Rap1 GTPases, which could explain the detachment of glial endfeet from the BM at the pial surface of the C3G^Emx1-KO^ cortex [20, 25]. To address this possibility, we stained cortical sections from E15 embryos with the VLA and 9EG7 anti-β1 integrin antibodies. While the VLA antibody visualizes the total level of β1 integrins, the 9EG7 antibody specifically detects activated β1 integrins. Staining with the VLA antibody did not reveal differences in the expression of β1 integrins in the VZ, the pial surface or blood vessels of the homozygous C3G^Emx1-KO^ cortex (Fig 4A and 4C). By contrast, the level of active β1 integrin was reduced in the mutant exclusively at the pial surface while the VZ remained unaffected (Fig 4A–4C). While the intensity profiles for VLA immunofluorescence signals (total β1 integrin) did not show obvious changes in the enrichment of β1 integrin at the pial surface, the signals for active β1 integrin (9EG7) were significantly reduced in the C3G^Emx1-KO^ cortex at the MZ indicating a loss of active β1 integrins in RGC endfeet. These results show that C3G is required for the activation of β1 integrins at the pial surface.

### C3G is required for the formation of axons and dendrites

**Fig 5:**
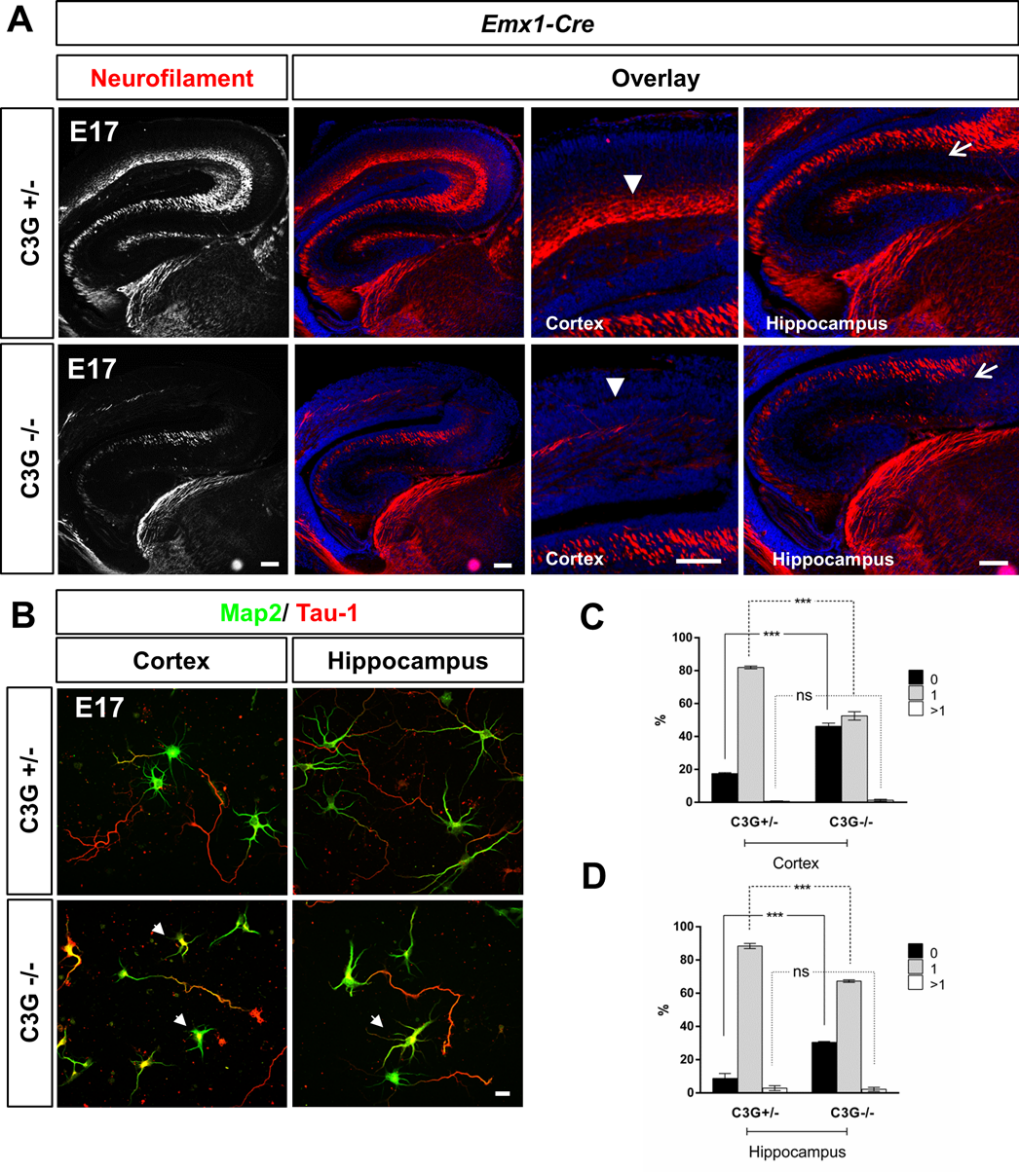
C3G is required for axon formation in the cortex and hippocampus. (A) Coronal sections from the caudal brain of heterozygous or homozygous C3G^Emx1-KO^ E17 embryos were stained with Hoechst 33342 (blue, nuclei) and an anti-NFM antibody (red) as a marker for axons. The mutant cortex shows an extensive loss of axons in the cortex (arrowheads) and hippocampus (arrows). A higher magnification is shown on the right (n ≥ 4 embryos from different litters for each genotype). Dorsal is to the top and medial to the right. (B) Neurons from the cortex or hippocampus of heterozygous or homozygous C3G^Emx1-KO^ embryos were stained at 3 d.i.v. with the Tau-1 (axons, red) and an anti-MAP2 (minor neurites, green) antibody. Unpolarized neurons without an axon are marked by arrowheads. (C, D) The percentage of unpolarized neurons without an axon (0, black), polarized neurons with a single axon (1, gray) and neurons with multiple axons (>1, white) is shown (n = 3 independent experiments, 100 neurons from each group, means ± s.e.m.; *** p≤0.001 compared to control determined by two-way ANOVA with Tukey’s multiple comparison test). Single confocal planes are shown. Scale bars are 100 μm (A) and 20 μm (B).

**Fig 6:**
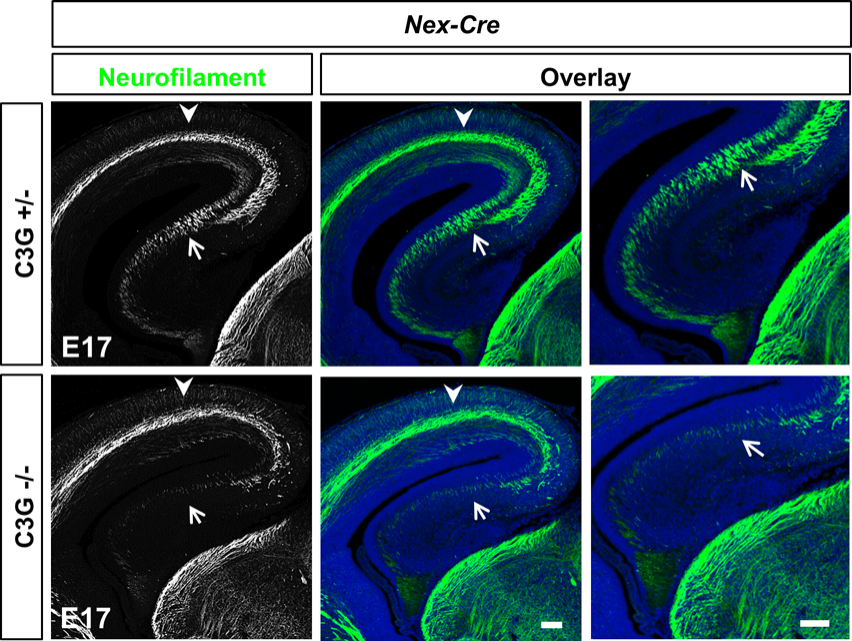
Selective loss of axons in the C3G^Nex-KO^ hippocampus. Coronal sections from heterozygous and homozygous C3G^Nex-KO^ E17 embryos were stained with an anti-NFM antibody (green) and Hoechst 33342 (blue). A marked loss of axons can be seen in the hippocampus (bottom, arrows) but not the cortex (top, arrowheads). A higher magnification of the hippocampus is shown in the rightmost panels. Dorsal is to the top and medial to the right. Single confocal planes are shown. Scale bars are 100 μm. Images are representative for 3 independent experiments with 3 embryos per genotype from different litters.

Previously, we have shown that Rap1 GTPases are required *in vivo* for the MTB transition and axon formation [24]. To analyze whether there are defects in axon formation also in the C3G^Emx1-KO^ mutant cortex, we stained sections from E17 brains with antibodies for neurofilament (NF) subunits as axonal markers. A severe defect in axon formation was observed in C3G^Emx1-KO^ embryos in the cortex and the hippocampus (Fig 5A) and persisted at P7 (S5B Fig). Only few axons extended above the VZ and, in some cases, axon bundles were also observed beneath the pia (S4 Fig). Staining with the pan-axonal marker SMI312 and NF light chain that marks only a subset of axons revealed the same defects in Rap1^Emx1-KO^ and C3G^Emx1-KO^ embryos (S5A Fig). Thus, C3G is required for axon formation by neurons derived from the dorsal telencephalic region of the developing neocortex. To determine if C3G is required in neurons we stained sections from the C3G^Nex-KO^ cortex with different anti-neurofilament antibodies. While no defect in axon formation was detectable in the C3G^Nex-KO^ cortex at E17 a significant defect was found in the hippocampus (Fig 6). Axonal staining was almost completely absent in the hippocampus while neurons could still be detected (S6 Fig). The same phenotype was observed in *Rap1a*^f/f^;*Rap1b*^f/f^;*Emx1*^Cre/+^ knockout mice [24]. These results indicate that C3G is required for axon formation in the cortex at a much earlier stage than in the hippocampus.

To better quantify the effects on axon formation, we cultured cortical and hippocampal neurons from E17 embryos. The neurons were fixed at 3 days in vitro (d.i.v.) and stained with anti-MAP2 and Tau-1 antibody as markers for minor neurites and axons, respectively. Cultures of cortical and hippocampal neurons from C3G^Emx1-KO^ embryos showed a significant reduction in the number of polarized neurons (cortex: 46 ± 2% unpolarized neurons; hippocampus: 30 ± 0.4%) compared to controls (cortex: 17 ± 1% unpolarized neurons; hippocampus: 9 ± 3%) (Fig 5B and 5C). The unpolarized neurons did not extend any Tau-1 positive neurite. These results confirm that C3G is required for polarization in both cortical and hippocampal neurons.

To investigate if C3G is also involved in the formation of dendrites we performed Golgi staining of the P7 cortex. Golgi staining showed severe defects in the formation of apical dendrites in the C3G^Emx1-KO^ (S7 Fig). Only 46 ± 2% of neurons possessed an apical dendrite compared to 86 ± 1% in heterozygous controls (S7A, S7B and S7D Fig). A significant proportion of these apical dendrites displayed a random orientation. In heterozygous *Rapgef1* knockouts, only 8 ± 0.2% of the apical dendrites deviate by more than ±15 degrees from the radial axis compared to 26 ± 4% in C3G^Emx1-KO^ brains (S7C Fig). These results show that C3G is required *in vivo* for the formation of both, axons and dendrites in the cortex. Since the apical dendrite develops from the leading process [8], the defect in dendrite formation could indicate a failure to polarize a leading process.

### C3G is required for two polarization events in cortical neurons

The presence of axons in the C3G^Nex-KO^ cortex in contrast to the almost complete loss in the C3G^Emx1-KO^ mutant raises the question at what stage of neuronal differentiation C3G is required. We have previously shown that Rap1 GTPases have to be inactivated early in multipolar neurons to interfere with neuronal polarity before the *Nex* promoter becomes active too late to affect axon formation [24]. To inactivate *Rapgef1* at an earlier stage and to delineate the temporal requirement more precisely, we directly analyzed neuronal migration in cortical slices by live cell imaging using different Cre expression vectors and the *ex vivo* electroporation of brains from E13.5 embryos. We first transfected the cortex of wild type or homozygous *Rapgef1*^flox/flox^ embryos with the plasmids *pEF-Cre*, *pEF-LPL-LynN-EGFP* and *pTα-LPL-H2B-mRFP*. Expression of Cre inactivates the floxed alleles and removes the stop cassette in the GFP expression vector to label these cells. The expression of LynN-EGFP and H2B-mRFP depends on the Cre-mediated removal of a stop cassette [7]. *pEF-Cre*, *pEF-LPL-LynN-EGFP* and *pTα-LPL-H2B-mRFP* drive Cre and GFP expression already in neuronal progenitors while H2B-mRFP marks neurons. Staining with an anti-C3G antibody confirmed the loss of C3G immunoreactivity in slices from *Rapgef1*^flox/flox^ embryos (S8A and S8B Fig).

**Fig 7:**
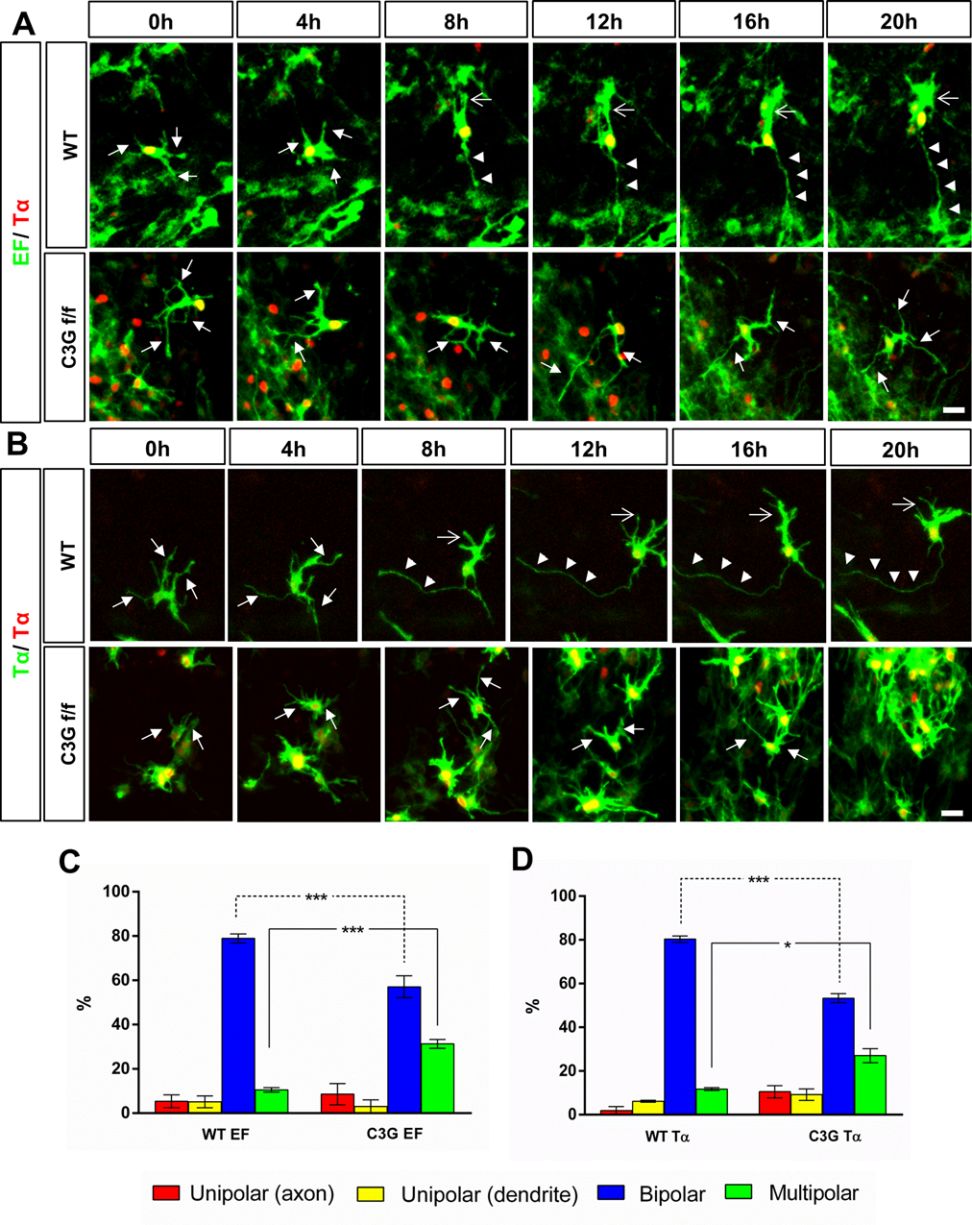
C3G is required in multipolar neurons for neuronal polarization. (A-B) Wild type (WT) or *Rapgef1*^flox/flox^ (C3G f/f) E13.5 brains were transfected by *ex vivo* electroporation with (A) *pEF-Cre*, *pEF-LPL-LynN-EGFP* and *pTα-LPL-H2B-RFP* or (B) *pTα-Cre*, *pTα-LPL-LynN-EGFP* and *pTα-LPL-H2B-RFP* to specifically inactivate the conditional alleles and label early post-mitotic neurons. Imaging was performed 30h after electroporation. Neurons from WT coronal slices first extend a long trailing process followed by a leading process. Slices from *Rapgef1*^flox/flox^ brains showed a significant number of neurons that remained multipolar and did not extend a trailing or a leading process after more than 20 h of imaging. (C, D) The percentage of cells that formed only a trailing axon (unipolar (only axon), red), only a leading process (unipolar (only leading process), yellow), that became bipolar (blue), or remained multipolar (green) after transfection of *pEF-Cre* (C) or *pTα-Cre* (D) at the end of the imaging period of 20 h (means ± SEM, ***p ≤ 0.001, *p ≤ 0.05 two-way ANOVA with Tukey’s multiple comparison test; number of bipolar or multipolar neurons from *Rapgef1*^flox/flox^ slices compared to control slices; n = 37 (wildtype; EF), n = 29 (*Rapgef1*^flox/flox^; EF) and n = 51 (wildtype; Tα), n = 45 (*Rapgef1*^flox/flox^; Tα) from 3 independent experiments that each included multiple slices from different animals, n indicates the total number of neurons analyzed in all experiments). The VZ is to the bottom and the pial surface to the top. Scale bars are 20 μm.

After transfection of *pEF-Cre*, most of the neurons became bipolar in wild type slices (79 ± 2%; n = 37 neurons) while only 11 ± 1% remained in the multipolar stage during the imaging time (S1 Video). By contrast, a large proportion of *Rapgef1* neurons remained in the multipolar phase (31 ± 2%; n = 29) even at 30 h after electroporation when control neurons already were polarized and had started their radial migration (Fig 7) (S2 Video). A small number of cells were unipolar and extended a leading process but no difference was found between wild type and *Rapgef1*^flox/flox^ slices for these unipolar cells.

The MTB transition was also blocked when the conditional knockout alleles were inactivated only in multipolar neurons using the combination of *pTα-Cre*, *pTα-LPL-LynN-EGFP* and *pTα-LPL-H2B-mRFP* [7, 24]. A significant number of *Rapgef1* neurons remained in the multipolar phase (27 ± 3%; n = 45) after transfection of *pTα-Cre* (Fig 7, S4 Video). By contrast, wild type neurons became bipolar and started their migration (80 ± 1%; n = 51) while only few (12 ± 1%,) remained in the multipolar stage during the imaging period (S3 Video). Thus, inactivating C3G in neurons with *pTα-Cre* caused the same phenotype at a similar frequency as that observed after transfection of *pEF-Cre* that is active already in RGCs. These experiments show that C3G is required for the formation of both axons and leading processes. Deletion of *Rapgef1* in multipolar neurons was sufficient to induce polarity defects. Thus, C3G is required in early multipolar neurons for both polarization steps.

## Discussion

We have shown previously that Rap1 GTPases are master regulators of polarity in the developing cortex [24]. In RGCs, they maintain cell polarity by regulating cadherin- and integrin-dependent adhesion. In addition, Rap1 GTPases are required in multipolar neurons for the MTB transition. Here we show that the Rap1 GEF C3G acts through integrins to promote the adhesion of RGC endfeet to the pial surface but is not required for the maintenance of AJs. In multipolar neurons, C3G is required for the MTB transition like the Rap1 GTPases. Conditional inactivation of *Rapgef1* in neuronal progenitors using *Emx1-Cre* results in the loss of axons and a disruption of cortical lamination. By contrast, we did not observe major defects in cortical development when *Rapgef1* was inactivated in bipolar neurons using *Nex*-*Cre*. This result is consistent with our previous analysis of *Rap1a*^f/f^;*Rap1b*^f/f^;*Nex-Cre* mice that did not show defects in neuronal polarity because the expression of Cre driven by the *Nex* promoter is too late to affect the MTB transition.

The defects in RGCs in the C3G^Emx1-KO^ cortex are less pronounced than in Rap1-deficient mutant [24] and RGCs that serve as substrate for neuronal migration still span the whole cortex. To address the question if C3G is required cell autonomously in neurons, we inactivated *Rapgef1* in multipolar neurons by the *ex vivo* electroporation of the cortex from Rapgef1^flox/flox^ embryos with the *pTα-Cre* vector [7, 24]. Live cell imaging of neuronal migration in cortical slices after conditional deletion in individual neurons showed that inactivation of Rapgef1 at an early time point results in a block of the MTB transition similar to the phenotype of Rap1-deficient multipolar neurons. Our results show that C3G is required *in vivo* in multipolar neurons for the specification of an axon and the initiation of radial migration by forming a leading process. In the *Nex-Cre* mediated knockout, axon formation is affected exclusively in the hippocampus but not in the cortex. This result indicates that C3G, similar to Rap1 GTPases [24], is required for axon formation at a later time point in hippocampal neurons probably because they remain in the multipolar phase of migration much longer before they become bipolar [34].

Unlike the *Rap1a*;*Rap1b* knockout, no defects in the formation of adherens junctions were detectable in C3G^Emx1-KO^ mice. However, the attachment of RGC endfeet to the pial BM, which is mediated by integrins, is disrupted. The overmigration of neurons through the BM that was visible as a cobblestone lissencephaly in the C3G^Emx1-KO^ resembles the phenotype of cortex-specific *Ptk2* (encoding FAK) and *Itgb1* (β1 integrin) knockouts [32, 33, 35, 36]. Unlike the C3G^Emx1-KO^ cortex, these mutants do not show defects in the formation of cortical layers with the exception of an invasion of the marginal zone by neurons. The absence of major RGC defects in the VZ of C3G^Emx1-KO^ compared to the loss of AJs in the Rap1-deficient cortex indicates that another GEF links Rap1 activity and N-cadherin function in the VZ. At least eight different Rap1 GEFs have been described [37, 38]. Among these, only PDZ-GEF1 (encoded by *Rapgef2*) and C3G have been shown to be required during cortical development [26, 28]. The C3G^Emx1-KO^ cortex does not show defects in the VZ while a *Rapgef2* knockout mediated by *Emx1-Cre* leads to the formation of an ectopic cortical mass beneath the normal cortex [28]. The *Rapgef2* knockout phenotype is very similar to that of αE-catenin, which also shows a heterotopic cortex [39]. This indicates that at least some aspects of the *Rapgef2* mutant phenotype may result from defects in AJs. Interestingly, the homotopic cortex has largely normal cortical layers and axon formation is not affected in the *Rapgef2* mutant. Thus, C3G and Rapgef2 appear to regulate different aspects of Rap1 function in RGCs. C3G regulates integrin-dependent adhesion in RGC endfeet while Rapgef2 may be required for the formation of AJs.

Recently, it was reported that Rapgef2 regulates Rap1 GTPases during the MTB transition [27]. Knockdown of Rapgef2 blocked axon formation and radial migration in the lower IZ. In contrast to these results, the conditional *Rapgef2*;*Emx1-Cre* mutant does not show major defects in axon formation [28]. However, this mutant was analyzed only in the adult brain. Therefore, it is possible that the knockdown of Rapgef2 by *in utero* electroporation results in a delay of axon extension but does not completely block it. In contrast to the *Rapgef2* knockout, the loss of C3G leads to a severe loss of axons that persists postnatally. PDZ-GEF1 itself is regulated by Rap1, which can establish a positive feedback loop [40, 41]. In neurons, C3G and PDZ-GEF1 may act sequentially as shown for neurotrophin induced neurite extension [41]. It will be interesting to test in the future how Rapgef2 acts in conjunction with C3G to regulate neuronal differentiation.

The defects in the formation of cortical layers in the C3G^Nex-KO^ resemble that of the *Dab1*;*Nex-Cre* knockout consistent with the idea that C3G acts downstream of Dab1 [42]. In both knockouts, cortical neurons invade the MZ with the consequence that a distinct layer I is missing [42]. An invasion of Cux1^+^ neurons into layer I was prominent in the C3G^Nex-KO^ cortex indicating that C3G is required in these neurons for the final stages of migration [20, 27].

Taken together our results show that the Rap1 GEF C3G is required in multipolar neurons for the MTB transition by promoting the formation of an axon and a leading process. While deletion of *Rapgef1* in neurons shows the same effects on polarity as the inactivation of *Rap1a*;*Rap1b*, it has a less severe phenotype in RGCs where it selectively affects the integrin-dependent adhesion to the basal lamina of the pial surface.

## Materials and Methods

### Mice

Mice were housed at four to five per cage with a 12-h light/dark cycle (lights on from 07:00 to 19:00 h) at constant temperature (23°C) with *ad libitum* access to food and water. Adult mice were euthanized by cervical dislocation, while neonates and adult rats were sacrificed by exposure to CO_2_ followed by decapitation. Embryos were placed immediately on ice followed by decapitation. All animal protocols were approved by the Veterinär- und Lebensmittelüberwachungsamt Münster. *Emx1*-*Cre* mice [29] were obtained from The Jackson Laboratory (Bar Harbor, Maine). *Nex*-*Cre* mice [31] were generously provided by Dr. Klaus-Armin Nave (MPI für Experimentelle Medizin Göttingen). All mouse strains were kept in a C57Bl/6 background. *Rapgef1*^flox/+^;*Emx1*^cre/cre^ mice were crossed with *Rapgef1*^flox/flox^ animals to obtain the *Rapgef1*^flox/+^;*Emx1*^Cre/+^ genotype as control or the *Rapgef1*^flox/flox^;*Emx1*^cre/+^ knockout. Similar crosses were done with the *Nex*-*Cre* line. For convenience, knockouts are referred to as C3G^Emx1-KO^ and C3G^Nex-KO^ (*Emx1*^cre/+^ or *Nex*^cre/+^).

Genotyping was done by PCR using the following primers. C3G: 5’- AGCCTGTTGG CAAGTTTGG-3’ and 5’-CTGATGGAGAACCTAGCTGTGG-3’. Emx1: WT1 5’-AAGGTGTGGTTCCAGAATCG-3’, WT2 5’- CTCTCCACCAGAAGGCTGAG-3’, TG1 5’-GCGGTCTGGCAGTAAAAACTATC-3’ and TG2 5’-GTGAAACAGCATTGCTGTCACTT-3’.Nex: 5’-GAGTCCTGGAATCAGTCTT TTTC-3’, 5’-AGAATGTGGAGTAGGGTGAC-3’, 5’-CCGCATAACCAGTGAAACAG-3’.

### Generation of Rapgef1 conditional knockout mouse

*Rapgef1* conditional knockout mice were generated by flanking exons 17–21 (Ensembl ENSMUSG00000039844) with LoxP sites. The targeting vector was generated using BAC clones from a C57Bl/6J RPCIB-731 BAC library and was transfected into TaconicArtemis C57BL/6N Tac embryonic stem (ES) cells. ES clones were isolated and analyzed by Southern blot for correct homologous recombination and absence of additional integration sides. Chimeric mice were generated from validated ES cells by blastocyst injection. Puromycin (PuroR) and Neomycin (NeoR) resistance cassettes were flanked by FRT sites and used for positive selection of clones after homologous recombination. The resistance cassettes were removed by crossbreeding to Flp recombinase-expressing transgenic mice. After backcrossing of offspring to C57/BL6J, *Rapgef1* LoxP/+ heterozygotes (*Rapgef1*^flox/+^) lacking PuroR and NeoR cassettes as well as the Flp transgene were bred to homozygosity and used to maintain the line. The presence of conditional and wild type *Rapgef1* alleles was detected using standard genotyping PCR. Cre-mediated deletion of exons 17–21 (4.8 kb) generates a loss-of-function allele of *Rapgef1* lacking the regions encoding the RasGEF N-domain together with a part of the CDC25 domain and by generating a frame-shift from exon 16 to all downstream exons.

### *Ex vivo* electroporation and live cell imaging

Brains from either wild type, *Rapgef1*^flox/flox^ E13.5 embryos were used for *ex vivo* electroporation. Briefly, plasmids were mixed with Fast Green dye (0.5%) and injected into the lateral ventricle. Embryos were transfected by 5 pulses with 54 V for 50 ms at 1 s intervals using the ECM-830 BTX square wave electroporator (BTX, Gentronic Inc). We used the plasmids *pTα-Cre* [7, 43], *pTα-LPL-LynN-EGFP* [7], *pEF-Cre* [44] and *pEF-LPL-LynN-EGFP* [44] to sparsely label cells. *pEF-Cre* is expressed in progenitors as well as in neurons and we analyzed cells that showed a transition from a multipolar (having multiple dynamic processes) to a bipolar morphology [8]. The formation of trailing axons and leading processes was analyzed as described previously [43, 45]. The neuron-specific *Tα1* promoter in the *pTα-LPL-H2B-mRFP* plasmid marks early postmitotic neurons that are still multipolar [24]. The brains were embedded in 3% low melting agarose (Biozym). 300 μm slices were cut using a vibratome (Leica), placed onto the membrane of a Millicell tissue culture inserts (0.4 μm, 30mm) (Millipore), and cultured at the air/liquid interface using neurobasal medium supplemented with B27, N2 in 35mm tissue culture dishes at 37°C, 5% CO_2_ and 40% O_2_ [46]. Imaging was performed in an incubation chamber at 37°C and 5% CO_2_ 24 h—30 h after electroporation using a Zeiss LSM 700 laser scanning confocal microscope (Carl Zeiss MicroImaging, Jena, Germany) equipped with the Zeiss ZEN Software (Carl Zeiss MicroImaging). Images were taken every 30 minutes for a period of 24–30 h. Supplementary videos are presented as maximum intensity projections of 10 stacks with a z-step size of 8–10μm. To determine the effect of *pEF-Cre* or *pTα-Cre* mediated deletion in *Rapgef1*^flox/flox^ slices, immunostaining for C3G was performed using anti-C3G antibody. Method is described earlier [24].

### Antibodies

For immunofluorescence, we used rabbit anti-C3G (Santa Cruz Biotechnology, H-300/sc-15359, 1:150), mouse anti-N-Cadherin (Abcam # ab98952, 1:200), mouse anti-Nestin (BD Biosciences #611658, 1:200), rabbit anti-NF medium chain (Abcam #ab64300, 1:200) or mouse anti-NF medium chain (2H3, DSHB, 1:4), rabbit anti-NF light chain (Cell Signaling #2837, 1:200), mouse anti-SMI 312 (Covance SMI-312R, 1:200), rabbit anti-Cux1 (Santa Cruz Biotechnology #sc13024, 1:150), rabbit anti-Tbr1 (Abcam #31940, 1:400), rabbit anti-Tbr2 (Abcam #23345, 1:400), rat anti-β1 integrin [very late antigen (VLA), Chemicon, #MAB1997, 1:200], rabbit anti-Calretinin (Millipore #AB5054, 1:1000), mouse anti-chondroitin sulfate (clone CS-56, Sigma #C8035, 1:100), rabbit anti-Tbr1 (Abcam #ab31940, 1:500), mouse Tau-1 (Chemicon #MAB3420; 1:500), mouse anti-MAP2 (Chemicon #AB5622; 1:1000), Hoechst 33342 (Molecular probes, 1:6000) and goat secondary antibodies labeled with Alexa 488 or 594 (Molecular Probes, 1:800). The rat 9EG7 (supernatant) antibody was provided by D. Vestweber (Max Planck Institute for Molecular Biomedicine, Münster, Germany). Images were taken on a Zeiss LSM 700 confocal microscope using the Zeiss ZEN software.

### Electron Microscopy

Electron microscopy was done as described before [24]. Briefly, E17 brains were fixed in modified Karnovsky’s fixative (4% PFA and 1% glutaraldehyde in PBS (pH7.4) overnight) followed by 3 washes in PBS. The brains were embedded in 3% agarose (in PBS) and 200μm sections were prepared using a vibratome (Leica VT1000S). Sections were fixed in modified Karnovsky’s in 0.1M sodium cacodylate buffer (Sigma), washed with 0.1M cacodylate buffer and post-fixed with 2% OsO_4_. Washed tissues were subsequently stained *en block* in 1% aqueous samarium triacetate (Sigma). After washing with double-distilled water, samples were dehydrated in a graded ethanol series, rinsed with propylenoxyde (Serva) and embedded in Epon resin (Serva). After polymerization (24 h at 60°C) ultrathin (~60–70 nm) sections were cut using an ultramicrotome with a diamond knife (Reichert). The slices were collected on Formvar coated grids and imaged using an EM410 electron microscope (Phillips). Images were collected with a side-mounted 5 Mpix CMOS camera and processed offline using Photoshop and Zoner PS16.

### Immunostaining and imaging of neuronal cultures

Cortical and hippocampal neurons were isolated and cultured as described previously [47], fixed at 3 d.i.v. with 4% paraformaldehyde (PFA)/15% sucrose in phosphate buffered saline (PBS) for 20 min and permeabilized with 0.01% Triton X-100/0.1% Na-Citrate/PBS for 10 min on ice. After three washes with PBS, fixed cells were blocked for 1 h at RT with 10% normal goat serum (NGS)/PBS and incubated with the primary and secondary antibodies (anti-MAP2 and Tau-1) in blocking buffer. A Zeiss LSM 700 confocal laser scanning microscope was used for imaging and single planes are displayed. Image analysis was done using ImageJ 1.48v (NIH) and Adobe Photoshop CS5. The stage of neuronal differentiation was determined according to published criteria [47]. The percentage of neurons was determined that are unpolarized (do not form any Tau-1 positive axon), that are polarized (form a single Tau-1 positive axon and multiple MAP2-positive minor neurites), and that form multiple Tau-1 positive axons.

### DiI tracing

E17 mouse brains were fixed with 4% PFA for 1 h at 4°C, embedded in 3% agarose and 200 μm coronal sections were cut using a vibratome. Solid DiI crystals (1,1’-dioctadecyl-3,3,3’,3’-tetramethylindocarbocyanine perchlorate; #D-282; Molecular Probes) were placed at the pial surface (anterograde) or at the ventricular surface of the cortex under a dissecting microscope. Slices were incubated in a humidity chamber for up to 72 h at 37°C and were analyzed using a Zeiss 700 laser scanning microscope.

### Histology and Cytology

Brains were isolated from embryos at E11, E13, E15, E17, P0, or P7, fixed in 4% PFA in PBS and cryoprotected in 20% sucrose/PBS solution overnight at 4°C. After one wash in PBS, the brains were then embedded in O.C.T. medium (Tissue-Tek) and frozen on dry ice. Alternatively, E17 brains were fixed with Carnoy’s reagent, dehydrated in xylene and embedded in paraffin. Coronal 12 μm sections were cut using a cryostat or microtome (Leica). Antigen-retrieval was performed by boiling sections in 10 mM sodium citrate buffer (with 0.05% Tween20), pH 6.0 in a microwave for 10 min at 650 watts. The sections were blocked with 1.5% NGS in PBS with 0.03% Triton X-100 for 1 h and stained with primary antibody prepared in blocking buffer overnight at 4°C. Sections were washed 3 times in PBS, 10 min each and subsequently treated with secondary antibodies for 2 h at room temperature. Sections were imaged using a Zeiss 700 confocal laser scanning microscope and single planes are displayed.

### Haematoxylin-eosin staining

E17 embryos were stained with haematoxylin and eosin (HE) using standard procedures. Briefly, the brains were fixed with Carnoy’s reagent, dehydrated in xylene followed by paraffin embedding. Sections (12 μm) were deparaffinized and rehydrated. The rehydrated sections were stained with Mayer’s Hemalum for 3 min, followed by 1 min wash in tap water, incubated in 0.5% HCl in 70% ethanol for 10 sec and washed again in tap water for 10 min. Freshly filtered 0.05% Eosin G was used for staining (1 min) followed by dehydration.

### Golgi Staining

Golgi staining was performed with freshly dissected P7 brains using the FD Rapid GolgiStain^TM^ Kit according to the manufacturer's instructions (FD NeuroTechnologies). After incubation, brains were embedded in 3% agarose and 150 μm sections were prepared using a vibratome. The sections were mounted on gelatin-coated slides, dried and developed using the provided reagent. Grayscale pictures of stained neurons were segmented using the threshold feature of ImageJ 1.48v (NIH).

### Statistics and Data analysis

Data are presented as means ± s.e.m. and were analyzed by Student’s t-test and/or two-way ANOVA with Tukey’s multiple comparison test as indicated in the figure legends (Prism 5, Version 5.00, GraphPad Software, La Jolla and Microsoft Excel 2007). For the analysis of integrins with the VLA-β1 and 9EG7 antibodies, the fluorescence intensity values in files in the LSM format were quantified by using the Plot Profile function in Image J 1.48v and analyzing the central area of the cortex from the VZ to the pial surface (distance: 641 pixels). The significance of differences in fluorescence intensity (arbitrary units) at each pixel position was calculated between means at each position from 3 independent stainings using Student’s t-test (* p≤0.05, **p≤0.01, or ***p≤0.001). Rose Plot analysis was performed using Rose.Net Freeware (Version 0.10., Todd.A Thompson Software).

## Supporting Information

S1 Fig Targeting strategy and validation of the *Rapgef1* conditional knockout. Exons 17–21 were flanked by LoxP sites, the selection markers by FRT (NeoR) and F3 (PuroR) sites, respectively. Cre-mediated recombination of exons 17–21 results in the inactivation of C3G.
(TIF)

S2 Fig Analysis of intermediate progenitors and defects in preplate splitting in C3G^Emx1-KO^ but not C3G^Nex-KO^ embryos. (A-B) Coronal section from E13 C3G^Emx1-KO^ heterozygous and homozygous mutants were stained with anti-Tbr2 antibody that specifically labels the IPs. (B) The quantification of Tbr2^+^ cells per 10^4^ μm^2^ does not show a significant difference in the number of IPs. (C) Coronal sections from E17 C3G^Emx1-KO^ and C3G^Nex-KO^ embryos and heterozygous controls were stained with antibodies for CSPGs (green) and calretinin (red). The presence of CSPG- and calretinin-positive cells in the SP (marked by arrowheads) and at the pial surface indicates that the preplate is split in C3G^Nex-KO^ embryos. The cortex of C3G^Emx1-KO^ embryos displays dispersed CSPG staining (arrow) due to lamination defects and inversion of CP (n = 3 independent experiments with 3 embryos per genotype from different litters. Dorsal is to the top. Single confocal planes are shown. Scale bars are 100 μm.
(TIF)

S3 Fig Loss of C3G immunoreactivity in C3G^Nex-KO^ mutants. (A) Coronal sections from the brain of heterozygous (+/-) or homozygous (-/-) C3G^Nex-KO^ E17 embryos were stained with an anti-C3G antibody (green). Note the loss of immunoreactivity specifically in the CP and IZ of the mutant cortex. (B) Coronal sections from the brain of heterozygous (+/-) or homozygous (-/-) C3G^Nex-KO^ E17 embryos were stained with Hoechst 33342, marking the cell nuclei. The pial surface in the C3G^Nex-KO^ shows an invasion of cells into layer I at the marginal zone (n = 3 independent experiments with 3 embryos per genotype from different litters). Dorsal is to the top. Single confocal planes are shown. MZ, marginal zone. The scale bar is 100 μm.
(TIF)

S4 Fig Defects in axon formation and RGC organization in C3G^Emx1-KO^ embryos. DiI tracing of axonal tracts and RGCs were performed in coronal 200 μm slices from E17 brains with the indicated genotypes by placing DiI crystals on the pial or ventricular surface. RGC organization was also disrupted with a premature termination of basal processes (arrows) in C3G^Emx1-KO^ Tracing also shows severe defects in axon formation (arrowheads). (n = 3 independent experiments with 3 embryos per genotype from different litters). Note the axonal projections underneath the pial surface in C3G^Emx1-KO^ embryos (arrowheads). Dorsal is to the top. Scale bars are 100 μm.
(TIF)

S5 Fig The loss of axons in C3G^Emx1-KO^ and C3G^Nex-KO^ mice persists after birth. (A) Coronal sections from E17 C3G^Emx1-KO^ and C3G^Nex-KO^ brains were stained using the pan-axonal marker SMI-312 and an anti-NFL antibody, which marks only a subpopulation of axons. Both axonal markers reveal the loss of axons in the cortex and the hippocampus of C3G^Emx1-KO^ embryos but only the hippocampus of C3G^Nex-KO^ embryos. Arrowheads mark cortical axons and arrows mark hippocampal axons. Dorsal is to the top and medial to the left. (B) Coronal sections from P7 mice with the indicated genotypes were stained with Hoechst 33342 (blue, nuclei) and an anti-NFM antibody (red) to mark axons. The loss of axons in the cortex and hippocampus of C3G^Emx1-KO^ mice can be still seen at P7. A higher magnification of the hippocampus is shown in the right panels. At least 3 independent brains from different litters were analyzed. Dorsal is to the top and medial to the left. Single confocal planes are shown. Scale bars are 100 μm.
(TIF)

S6 Fig Loss of axons but not neurons in the hippocampus C3G^Emx1-KO^ and C3G^Nex-KO^ embryos. Coronal sections from the hippocampal region of E17 C3G^Emx1-KO^ and C3G^Nex-KO^ embryos and heterozygous controls (*Rapgef1*^flox/+^;Emx1^Cre/+^) were stained with anti-NeuN (neuronal marker, green) and an anti-NFM antibody (red). Staining for NeuN showed that the loss of axons does not result from an absence of neurons. Images are representative for 3 independent experiments with 3 embryos per genotype from different litters. Single confocal planes are shown. Scale bars are 100 μm.
(TIF)

S7 Fig C3G^Emx1-KO^ brains show defects in apical dendrite formation. (A) Golgi staining of 150 μm sections from the cortex of P7 wild type and C3G^Emx1-KO^ mice shows severe defects in apical dendrite formation. (B) Representative examples of neurons demonstrate that apical dendrites are lost or randomly orientated in C3G^Emx1-KO^ brains. (C) A rose plot displays the angle of apical dendrites relative to the horizontal axis in C3G cortices. Analysis for C3G^Emx1-KO^ neurons (C3G-/-) shows significantly higher number of neurons, which possess misoriented apical dendrites in comparison to heterozygous controls (C3G+/-). (D) The percentage of neurons with radially oriented dendrites (+), without apical dendrites (-) and with randomly oriented apical dendrites (misoriented) is shown (n = 3 independent experiments, with 3 embryos per genotype from different litters (control: 80, 133, 129 neurons quantified, C3G^Emx1-KO^: 112, 98, 208 neurons; means ± s.e.m.; *** p≤0.001 compared to control determined by two-way ANOVA with Tukey’s multiple comparison test). Dorsal is to the top. Scale bars are 100 μm.
(TIF)

S8 Fig Loss of C3G after conditional deletion in cortical slices by *ex vivo* electroporation. The cortex of E13.5 wild type (+/+) or *Rapgef1*^flox/flox^ (C3G f/f) E13.5 embryos was transfected by *ex vivo* electroporation with *pTα-Cre* and *pTα-LPL-LynN-EGFP* (A) to inactivate the conditional alleles and label early post-mitotic neurons. 40 h after electroporation, slices were fixed, 20 μm sections prepared and stained with an anti-C3G antibody. The position (panels on the left) and outline of GFP^+^ cells (green) are indicated (dotted line). Note that C3G immunoreactivity (red) was detectable mainly at the cell periphery. Transfected cells showed a marked reduction in immunoreactivity in comparison to the surrounding, non-transfected tissue. (B) A line scan across the soma of the transfected cells at the position indicated by a white line in (A) confirms the loss of C3G (n = 3 independent experiments that each included multiple slices from different animals). Single confocal planes are shown. Scale bars are 10 μm.
(TIF)

S1 Video E13.5 wild type brains were transfected by *ex vivo* electroporation with *pEF-Cre*, *pEF-LPL-LynN-EGFP*, and *pTα-LPL-H2B-RFP*. Neuronal migration was analyzed in cortical slices by live cell imaging beginning at 30 h after transfection (t = 0 h). The movie shows a multipolar neuron that forms a ventrally directed trailing axon (t = 8 h) followed by formation of a leading process (t = 12 h) and the transition to a bipolar morphology.
(MOV)

S2 Video E13.5 *Rapgef1*^flox/flox^ brains were transfected by *ex vivo* electroporation with *pEF-Cre*, *pEF-LPL-LynN-EGFP*, and *pTα-LPL-H2B-RFP*. Neuronal migration was analyzed in cortical slices by live cell imaging beginning at 30 h after transfection (t = 0). The movie shows a multipolar neuron that fails to polarize and form a trailing axon or a radially oriented leading process, even after 20 h of imaging.
(MOV)

S3 Video E13.5 wild type brains were transfected by *ex vivo* electroporation with *pTα-Cre*, *pTα-LPL-LynN-EGFP*, and *pTα-LPL-H2B-RFP*. Neuronal migration was analyzed in cortical slices by live cell imaging beginning at 30 h after transfection (t = 0 h). The movie shows a multipolar neuron that tangentially extends out a trailing axon (t = 8 h) followed by formation of a leading process (t = 12–16 h) and the transition to a bipolar morphology.
(MOV)

S4 Video E13.5 *Rapgef1*^flox/flox^ brains were transfected by *ex vivo* electroporation with *pTα-Cre*, *pTα-LPL-LynN-EGFP*, and *pTα-LPL-H2B-RFP*. Neuronal migration was analyzed in cortical slices by live cell imaging beginning at 30 h after transfection (t = 0). The movie shows a multipolar neuron that fails to polarize and form a trailing axon or a radially oriented leading process, even after 20 h of imaging.
(MOV)
