# Supplementary figures and images for: C3G/Rapgef1 Is Required in Multipolar Neurons for the Transition to a Bipolar Morphology during Cortical Development
Source: PLoS One. 2016 Apr 25;11(4):e0154174. doi: 10.1371/journal.pone.0154174 (PMC4844105; doi:10.1371/journal.pone.0154174)

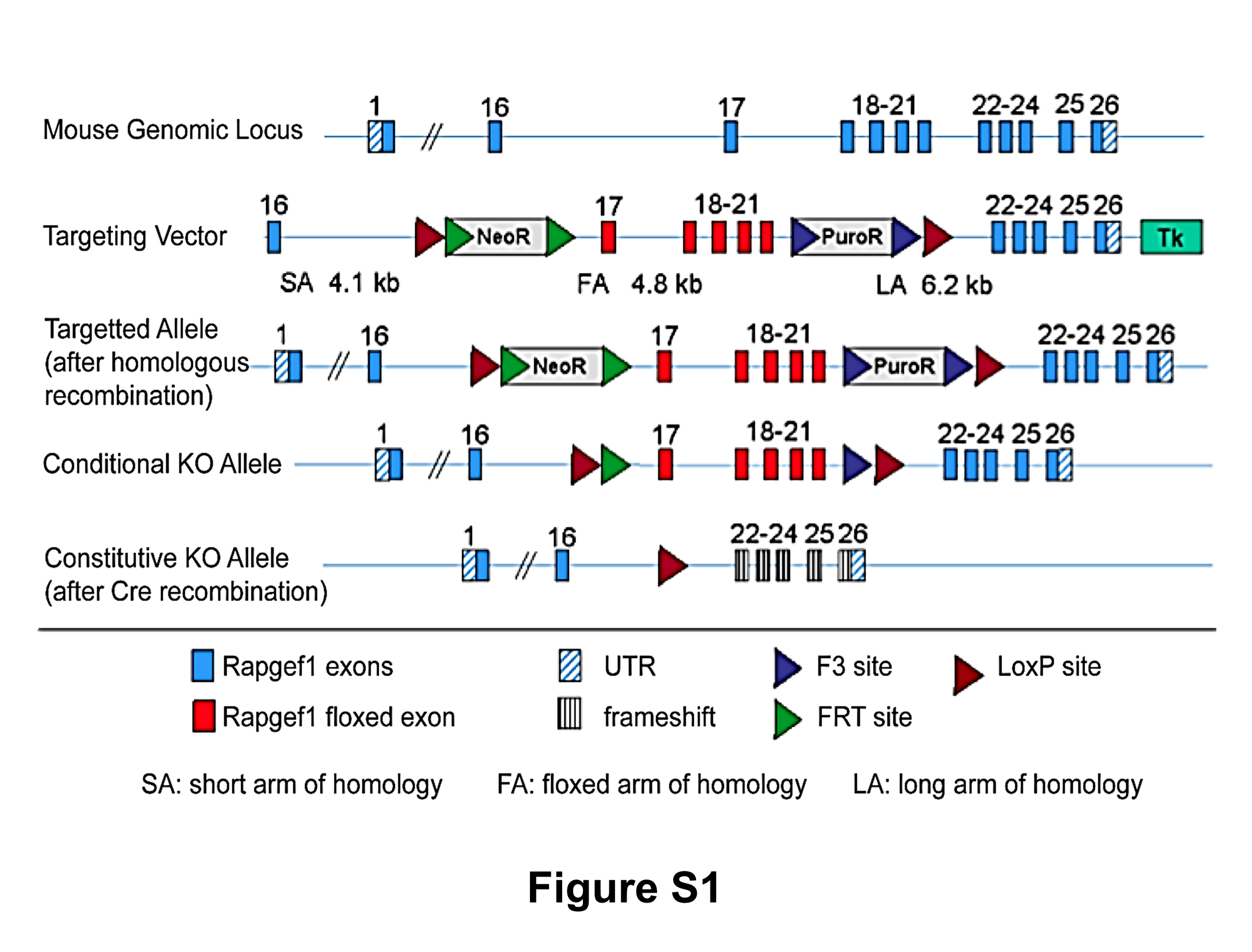

Supplement: S1 Fig — Exons 17–21 were flanked by LoxP sites, the selection markers by FRT (NeoR) and F3 (PuroR) sites, respectively. Cre-mediated recombination of exons 17–21 results in the inactivation of C3G. (TIF) [file pone.0154174.s001.tif]

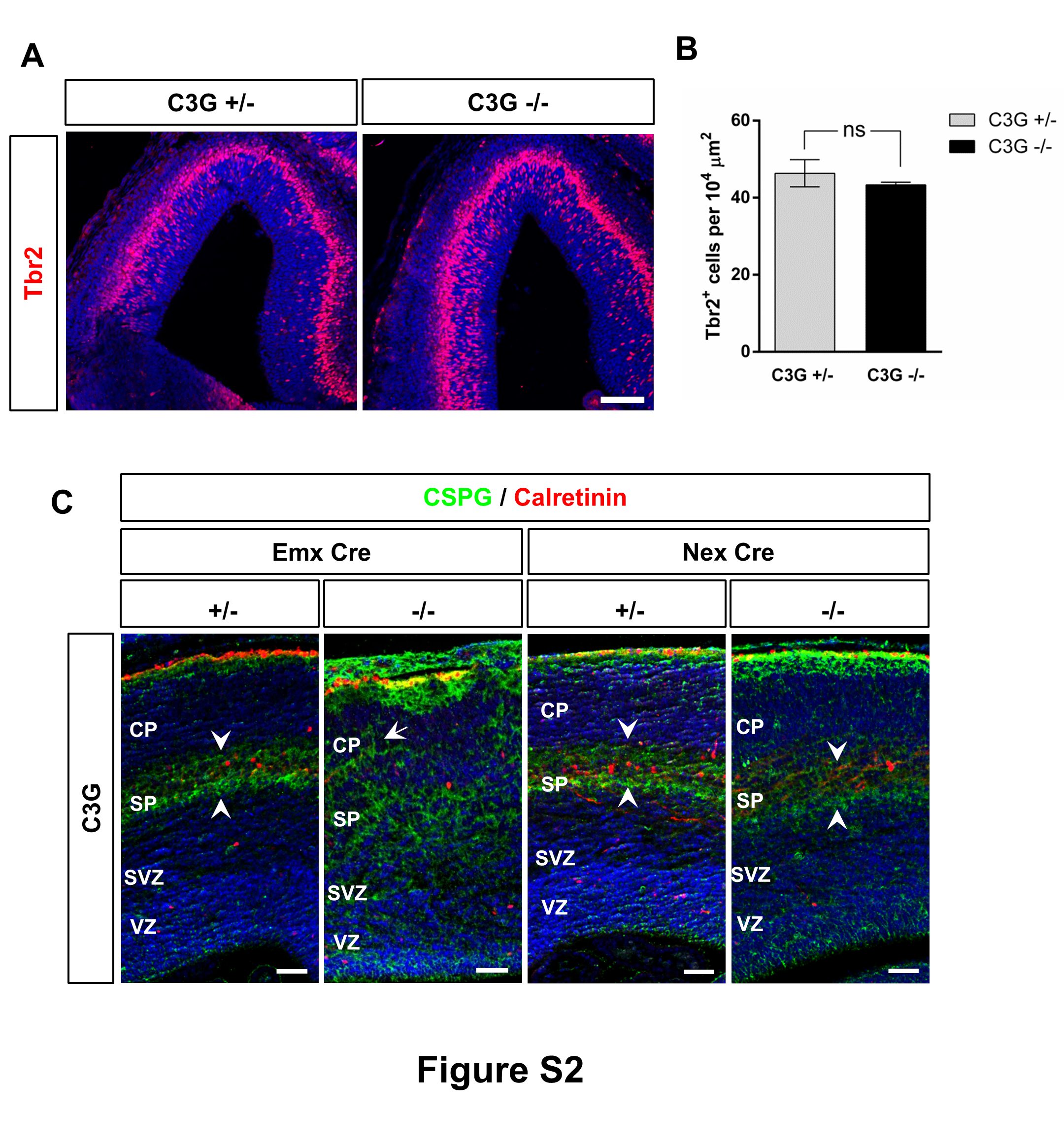

Supplement: S2 Fig — (A-B) Coronal section from E13 C3GEmx1-KO heterozygous and homozygous mutants were stained with anti-Tbr2 antibody that specifically labels the IPs. (B) The quantification of Tbr2+ cells per 104 μm2 does not show a significant difference in the number of IPs. (C) Coronal sections from E17 C3GEmx1-KO and C3GNex-KO embryos and heterozygous controls were stained with antibodies for CSPGs (green) and calretinin (red). The presence of CSPG- and calretinin-positive cells in the SP (marked by arrowheads) and at the pial surface indicates that the preplate is split in C3GNex-KO embryos. The cortex of C3GEmx1-KO embryos displays dispersed CSPG staining (arrow) due to lamination defects and inversion of CP (n = 3 independent experiments with 3 embryos per genotype from different litters. Dorsal is to the top. Single confocal planes are shown. Scale bars are 100 μm. (TIF) [file pone.0154174.s002.tif]

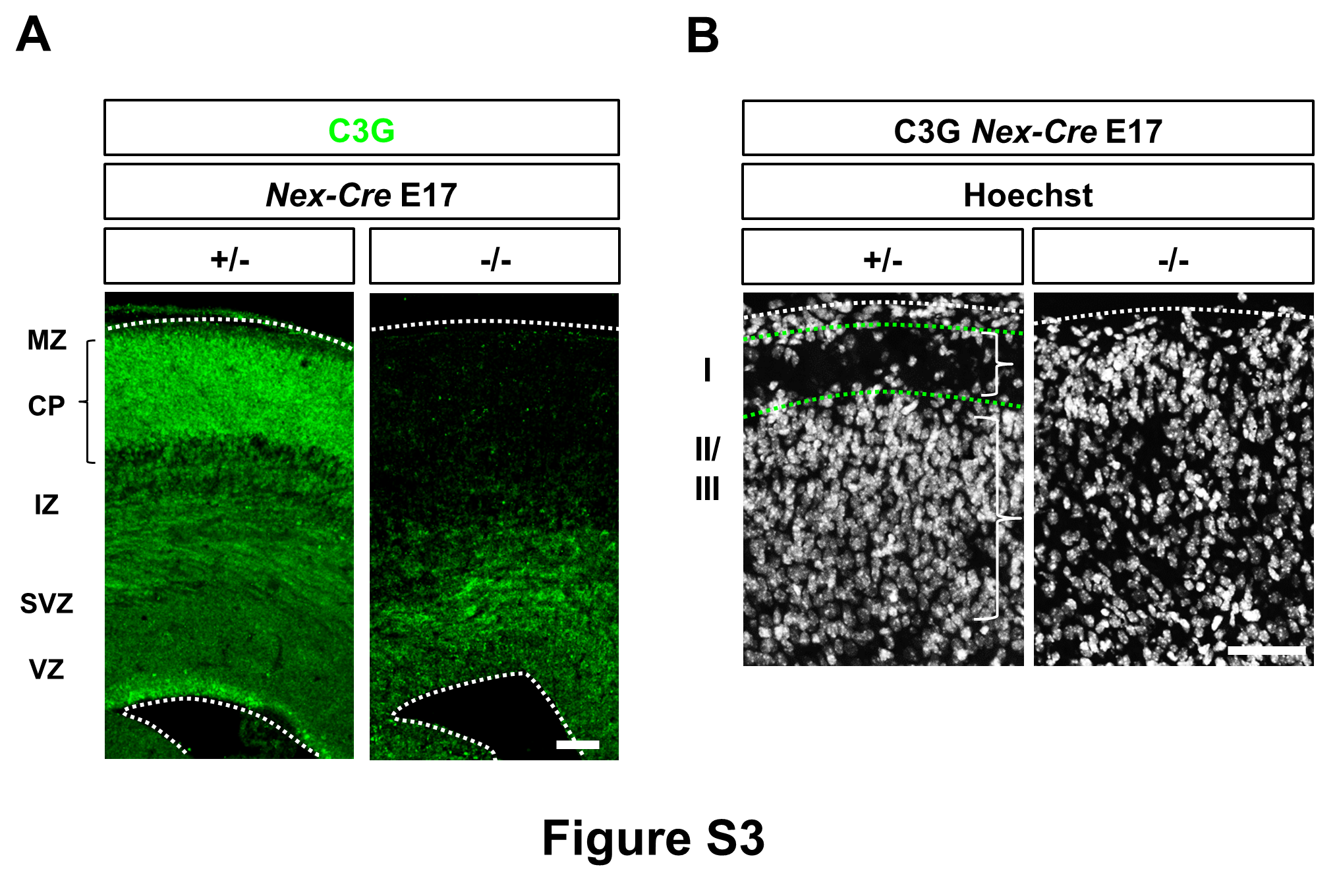

Supplement: S3 Fig — (A) Coronal sections from the brain of heterozygous (+/-) or homozygous (-/-) C3GNex-KO E17 embryos were stained with an anti-C3G antibody (green). Note the loss of immunoreactivity specifically in the CP and IZ of the mutant cortex. (B) Coronal sections from the brain of heterozygous (+/-) or homozygous (-/-) C3GNex-KO E17 embryos were stained with Hoechst 33342, marking the cell nuclei. The pial surface in the C3GNex-KO shows an invasion of cells into layer I at the marginal zone (n = 3 independent experiments with 3 embryos per genotype from different litters). Dorsal is to the top. Single confocal planes are shown. MZ, marginal zone. The scale bar is 100 μm. (TIF) [file pone.0154174.s003.tif]

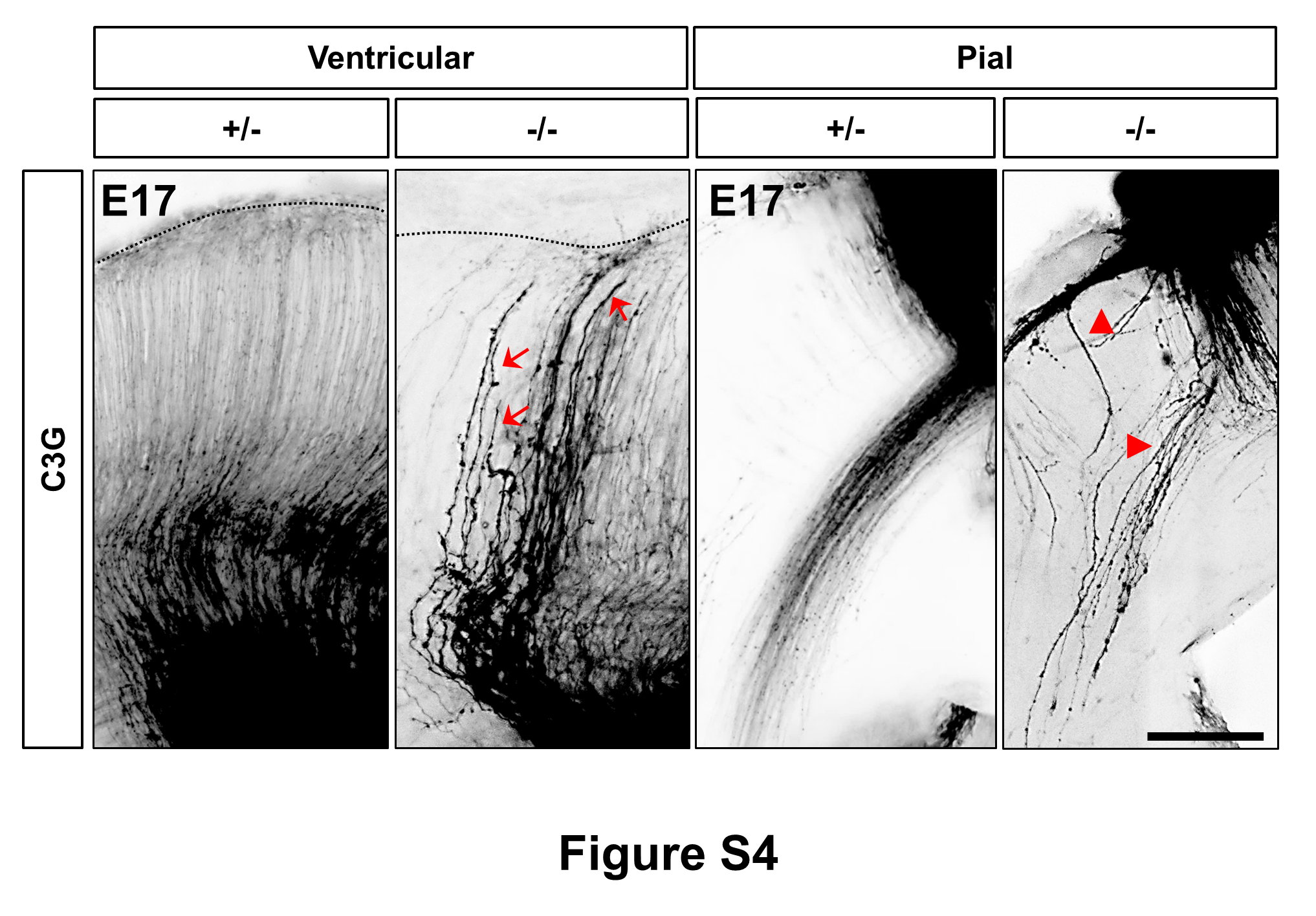

Supplement: S4 Fig — DiI tracing of axonal tracts and RGCs were performed in coronal 200 μm slices from E17 brains with the indicated genotypes by placing DiI crystals on the pial or ventricular surface. RGC organization was also disrupted with a premature termination of basal processes (arrows) in C3GEmx1-KO Tracing also shows severe defects in axon formation (arrowheads). (n = 3 independent experiments with 3 embryos per genotype from different litters). Note the axonal projections underneath the pial surface in C3GEmx1-KO embryos (arrowheads). Dorsal is to the top. Scale bars are 100 μm. (TIF) [file pone.0154174.s004.tif]

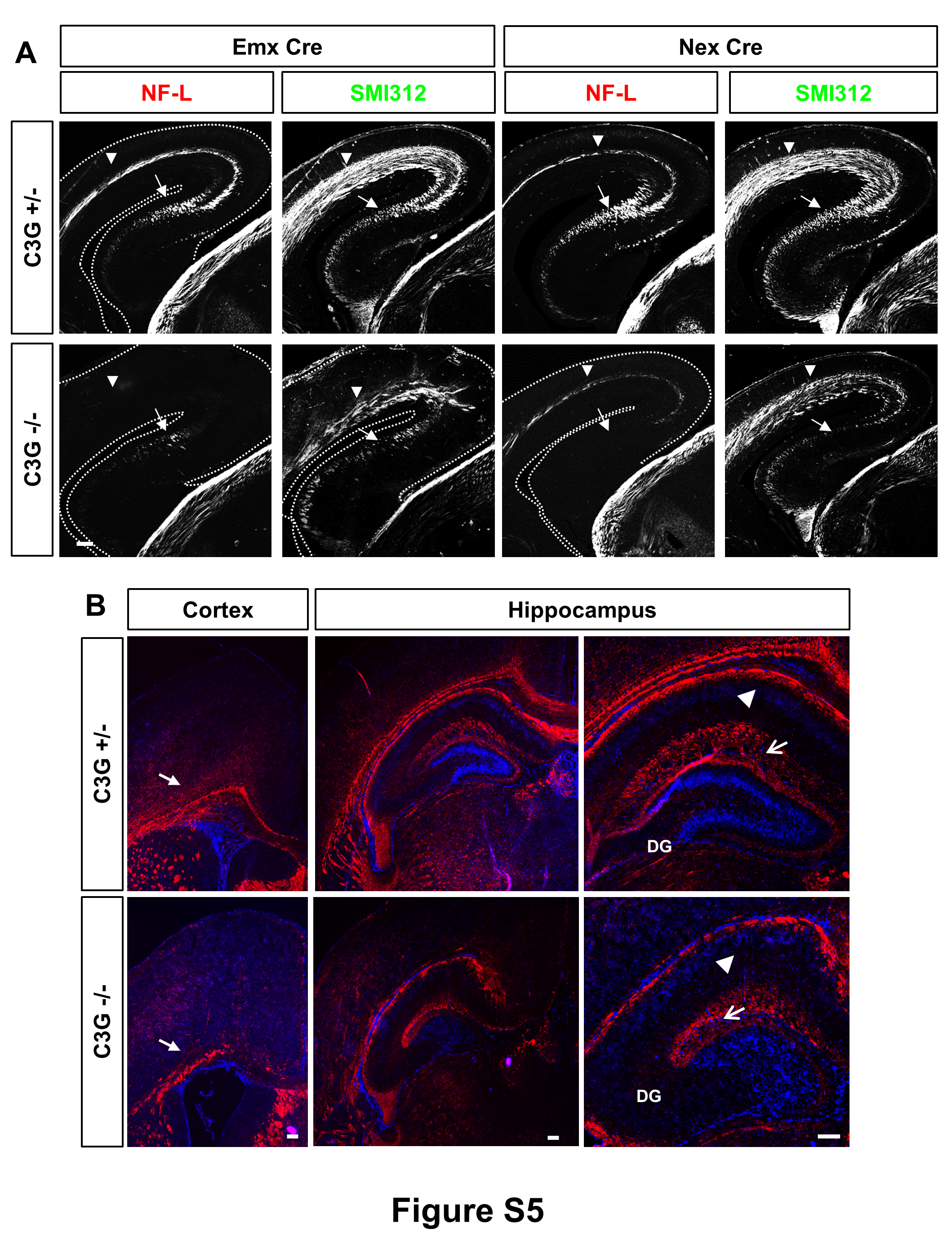

Supplement: S5 Fig — (A) Coronal sections from E17 C3GEmx1-KO and C3GNex-KO brains were stained using the pan-axonal marker SMI-312 and an anti-NFL antibody, which marks only a subpopulation of axons. Both axonal markers reveal the loss of axons in the cortex and the hippocampus of C3GEmx1-KO embryos but only the hippocampus of C3GNex-KO embryos. Arrowheads mark cortical axons and arrows mark hippocampal axons. Dorsal is to the top and medial to the left. (B) Coronal sections from P7 mice with the indicated genotypes were stained with Hoechst 33342 (blue, nuclei) and an anti-NFM antibody (red) to mark axons. The loss of axons in the cortex and hippocampus of C3GEmx1-KO mice can be still seen at P7. A higher magnification of the hippocampus is shown in the right panels. At least 3 independent brains from different litters were analyzed. Dorsal is to the top and medial to the left. Single confocal planes are shown. Scale bars are 100 μm. (TIF) [file pone.0154174.s005.tif]

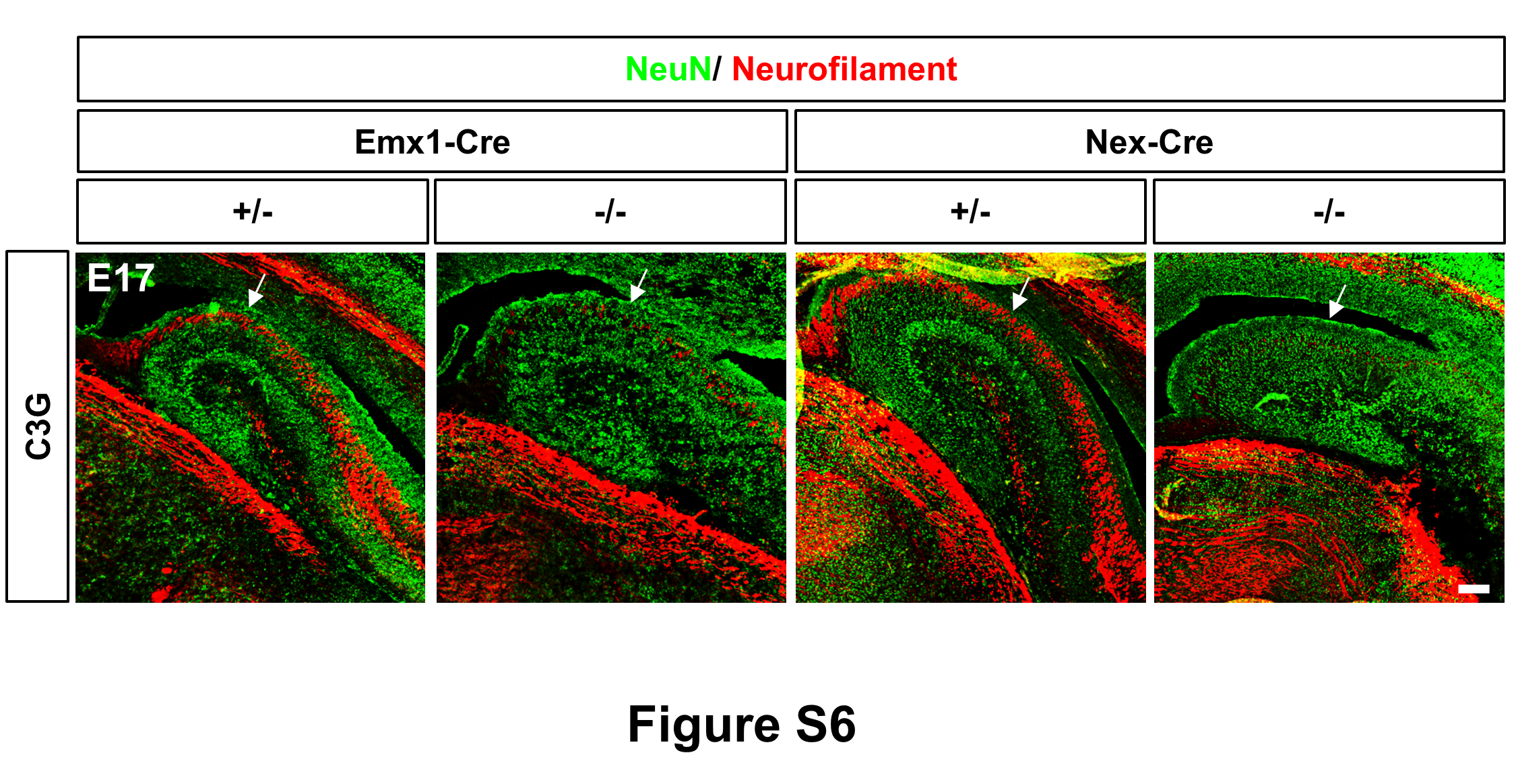

Supplement: S6 Fig — Coronal sections from the hippocampal region of E17 C3GEmx1-KO and C3GNex-KO embryos and heterozygous controls (Rapgef1flox/+;Emx1Cre/+) were stained with anti-NeuN (neuronal marker, green) and an anti-NFM antibody (red). Staining for NeuN showed that the loss of axons does not result from an absence of neurons. Images are representative for 3 independent experiments with 3 embryos per genotype from different litters. Single confocal planes are shown. Scale bars are 100 μm. (TIF) [file pone.0154174.s006.tif]

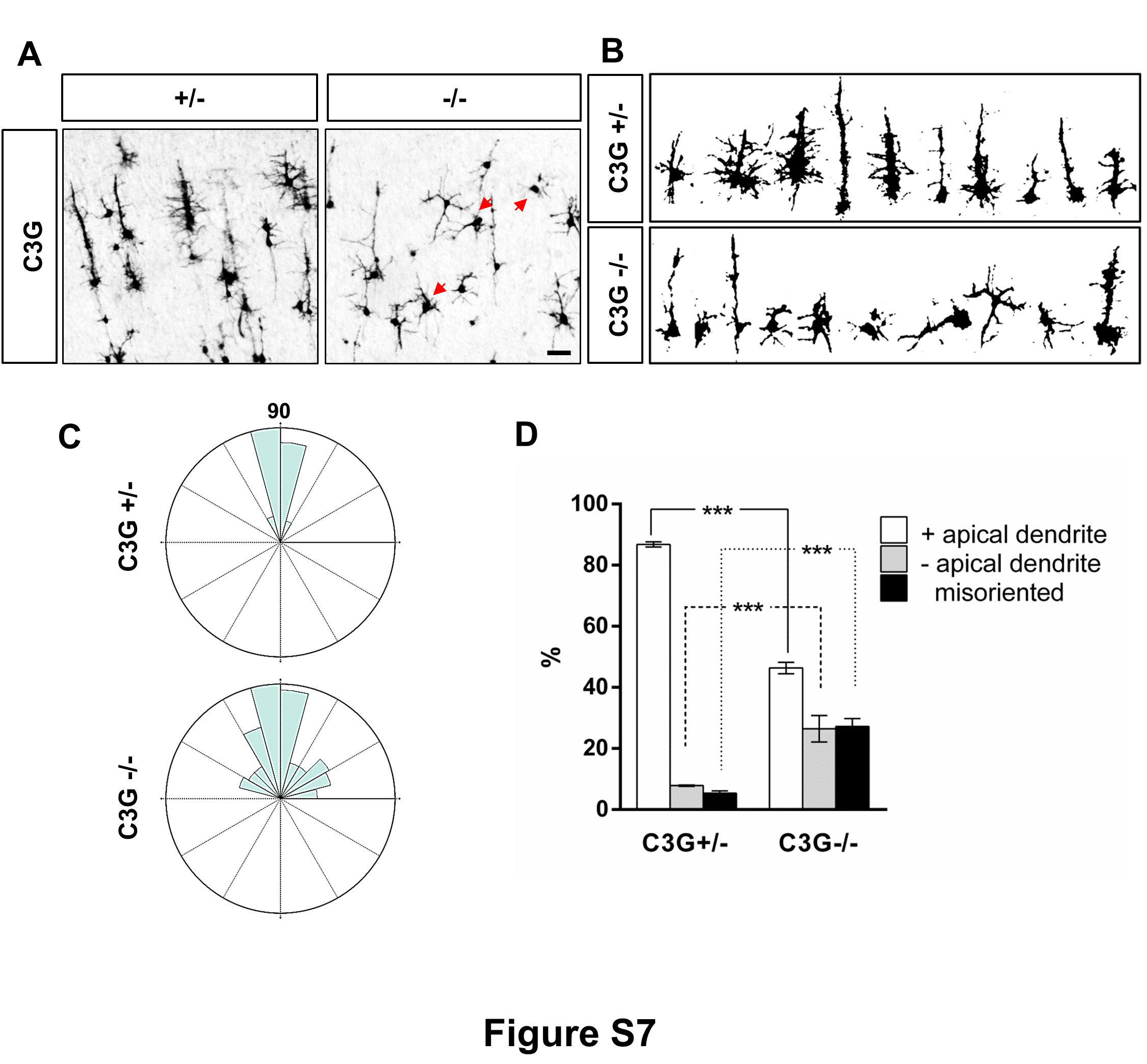

Supplement: S7 Fig — (A) Golgi staining of 150 μm sections from the cortex of P7 wild type and C3GEmx1-KO mice shows severe defects in apical dendrite formation. (B) Representative examples of neurons demonstrate that apical dendrites are lost or randomly orientated in C3GEmx1-KO brains. (C) A rose plot displays the angle of apical dendrites relative to the horizontal axis in C3G cortices. Analysis for C3GEmx1-KO neurons (C3G-/-) shows significantly higher number of neurons, which possess misoriented apical dendrites in comparison to heterozygous controls (C3G+/-). (D) The percentage of neurons with radially oriented dendrites (+), without apical dendrites (-) and with randomly oriented apical dendrites (misoriented) is shown (n = 3 independent experiments, with 3 embryos per genotype from different litters (control: 80, 133, 129 neurons quantified, C3GEmx1-KO: 112, 98, 208 neurons; means ± s.e.m.; *** p≤0.001 compared to control determined by two-way ANOVA with Tukey’s multiple comparison test). Dorsal is to the top. Scale bars are 100 μm. (TIF) [file pone.0154174.s007.tif]

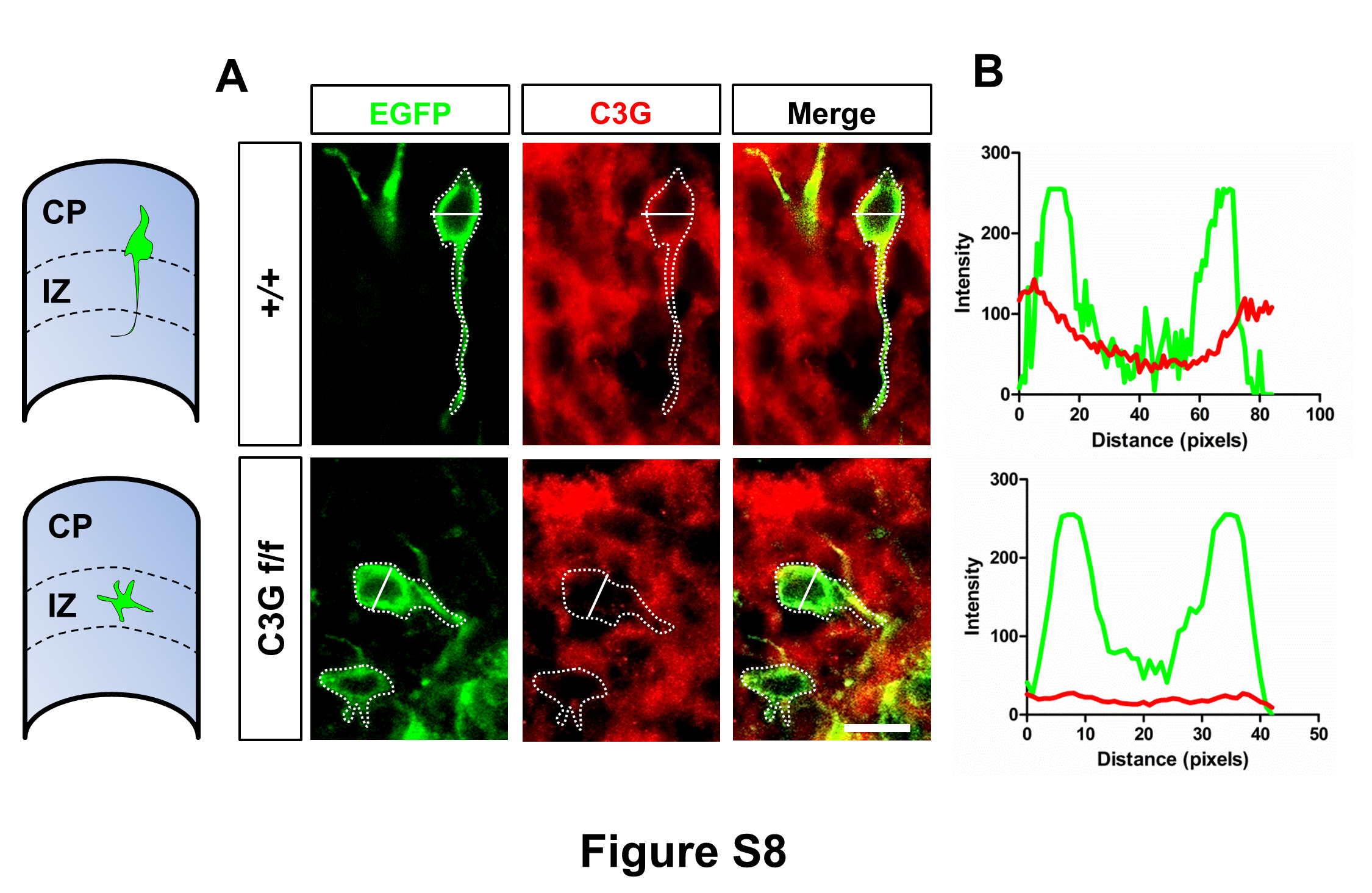

Supplement: S8 Fig — The cortex of E13.5 wild type (+/+) or Rapgef1flox/flox (C3G f/f) E13.5 embryos was transfected by ex vivo electroporation with pTα-Cre and pTα-LPL-LynN-EGFP (A) to inactivate the conditional alleles and label early post-mitotic neurons. 40 h after electroporation, slices were fixed, 20 μm sections prepared and stained with an anti-C3G antibody. The position (panels on the left) and outline of GFP+ cells (green) are indicated (dotted line). Note that C3G immunoreactivity (red) was detectable mainly at the cell periphery. Transfected cells showed a marked reduction in immunoreactivity in comparison to the surrounding, non-transfected tissue. (B) A line scan across the soma of the transfected cells at the position indicated by a white line in (A) confirms the loss of C3G (n = 3 independent experiments that each included multiple slices from different animals). Single confocal planes are shown. Scale bars are 10 μm. (TIF) [file pone.0154174.s008.tif]
